# Using AI-Based Evolutionary Algorithms to Elucidate Adult Brain Tumor (Glioma) Etiology Associated with IDH1 for Therapeutic Target Identification

**DOI:** 10.3390/cimb44070206

**Published:** 2022-07-02

**Authors:** Caitríona E. McInerney, Joanna A. Lynn, Alan R. Gilmore, Tom Flannery, Kevin M. Prise

**Affiliations:** 1 Patrick G. Johnson Centre for Cancer Research, Queen’s University Belfast, 97 Lisburn Rd, Belfast BT9 7AE, Northern Ireland, UK; jlynn10@qub.ac.uk (J.A.L.); alan.gilmore.holywood@googlemail.com (A.R.G.); k.prise@qub.ac.uk (K.M.P.); 2 Department of Neurosurgery, Royal Victoria Hospital, Belfast Health & Social Care Trust, Belfast BT9 7AB, Northern Ireland, UK; tom.flannery@belfasttrust.hscni.net

**Keywords:** glioblastoma, brain cancer, glioma, biomarker, artificial intelligence, evolutionary algorithm, isocitrate dehydrogenase 1, TCGA

## Abstract

Adult brain tumors (glioma) represent a cancer of unmet need where standard-of-care is non-curative; thus, new therapies are urgently needed. It is unclear whether isocitrate dehydrogenases (IDH1/2) when not mutated have any role in gliomagenesis or tumor growth. Nevertheless, IDH1 is overexpressed in glioblastoma (GBM), which could impact upon cellular metabolism and epigenetic reprogramming. This study characterizes IDH1 expression and associated genes and pathways. A novel biomarker discovery pipeline using artificial intelligence (evolutionary algorithms) was employed to analyze IDH-wildtype adult gliomas from the TCGA LGG-GBM cohort. Ninety genes whose expression correlated with IDH1 expression were identified from: (1) All gliomas, (2) primary GBM, and (3) recurrent GBM tumors. Genes were overrepresented in ubiquitin-mediated proteolysis, focal adhesion, mTOR signaling, and pyruvate metabolism pathways. Other non-enriched pathways included O-glycan biosynthesis, notch signaling, and signaling regulating stem cell pluripotency (PCGF3). Potential prognostic (TSPYL2, JAKMIP1, CIT, TMTC1) and two diagnostic (MINK1, PLEKHM3) biomarkers were downregulated in GBM. Their gene expression and methylation were negatively and positively correlated with IDH1 expression, respectively. Two diagnostic biomarkers (BZW1, RCF2) showed the opposite trend. Prognostic genes were not impacted by high frequencies of molecular alterations and only one (TMTC1) could be validated in another cohort. Genes with mechanistic links to IDH1 were involved in brain neuronal development, cell proliferation, cytokinesis, and O-mannosylation as well as tumor suppression and anaplerosis. Results highlight metabolic vulnerabilities and therapeutic targets for use in future clinical trials.

## 1. Introduction

Although regarded as “rare”, primary brain tumors (gliomas) are in fact the most common cause of cancer-related deaths in people under the age of 40 years [1]. The most prevalent and malignant type of primary brain tumor is a central nervous system (CNS) WHO Grade IV Glioblastoma (GBM) IDH-wildtype. GBM was formerly designated as either IDH-wildtype or IDH-mutant; however, the latter subtype has since been reclassified as Grade IV Astrocytoma IDH-mutant [2]. GBM accounts for 49.1% of all primary malignant brain and CNS tumors in adults [3]. Despite treatment, GBM has one of the lowest survival rates of all cancers [3]. Standard-of-care for GBM has not changed significantly since 2005. Therapy is non-curative and involves surgery (maximal safe tumor resection), radiotherapy, and alkylating chemotherapy with temozolomide (TMZ) [4]. New treatments trialed for GBM have all failed to markedly improve patient survival [5]. This is due in part to the complex biological characteristics of GBM and the diffuse nature of infiltration at the time of first presentation. Whilst a gross total or macroscopic resection is possible, this is not a complete microscopic resection. Thus, any post-operative remaining tumor volume inevitably leads to recurrence. Surgical resection is improving, however, with the wide adoption of intra-operative real-time tumor visualization using fluorescence [6]. Secondly, brain tumors are difficult to treat due to the blood–brain barrier (BBB), which separates the brain from the general blood circulation. Most therapeutic agents cannot diffuse across the BBB in sufficient quantities to achieve the free brain-to-plasma concentration ratios required for clinical efficacy [7]. Lastly, gliomas exhibit high levels of inter- and intra-tumor heterogeneity, including variability at the functional level [8,9,10,11]. Consequently, susceptibility of the tumor to treatment is uneven, leading to recurrence driven by radioresistant glioma stem cell populations [12]. Improved knowledge of glioma tumorigenesis has led to more precise tumor classification and personalized therapies; however, patient outcomes have not significantly improved. Thus, glioma remains a cancer of unmet need, and new targeted therapies are urgently needed.

In cancers including glioma, altered genes in metabolic pathways are key drivers of disease progression, providing the energy and building blocks for tumor cells to grow [13,14]. Amongst these are the isocitrate dehydrogenases (IDH), enzymes that are involved in cellular metabolism and DNA repair. As part of the citric acid cycle (TCA), IDH1 and IDH2/3 catalyze the conversion of isocitrate to alpha-ketoglutarate (also known as 2-oxoglutarate) in the cytoplasm and mitochondrial matrix, respectively. This cycle is completed by the transfer of electrons from NAD+ to NADPH. Alpha-ketoglutarate (α-KG) also enters into the TCA cycle as the metabolite of glutamine, whose utilization is enhanced to provide energy for cancer as part of aerobic glycolysis (i.e., the Warburg effect). Glioma tumors that are IDH-mutant and IDH-wildtype are biologically distinct, differing in their chromatin structure, gene expression, disease development and progression, and even prognosis, where IDH-wildtype status is associated with worse overall survival [15]. For IDH-mutant tumors, mutations in the active site of IDH1 (R132) and IDH2 (R172, R140) occur early and are maintained during glioma disease progression. IDH1/2 mutations cause the conversion of α-KG to the oncometabolite, D-2-hydroxyglutarate (D-2-HG) [16]. Accumulation of D-2-HG prevents DNA and histone demethylation and impairs cell differentiation and DNA repair, which all contribute to tumorigenesis in the IDH-mutant glioma phenotype [17,18]. It is not clear if IDH-wildtype (i.e., not mutated) has a role in gliomagenesis; however, IDH1 overexpression has been demonstrated in silico [10,19,20]. This pattern has also been confirmed in vivo using immunohistochemical analysis of protein [19]. In vivo studies have linked IDH1 to disease progression in primary GBM IDH-wildtype [19,20]. Inhibition of IDH1 using short hairpin (sh)RNAs or chemical molecules hindered GBM cell growth in vivo and extended the survival of xenograft mice models [19]. Tumor progression and resistance to cell death are potentially promoted by IDH1 via its efficient fatty acid synthesis and ROS scavenging activities [21]. In IDH-wildtype gliomas, α-KG is an obligatory co-factor of dioxygenase enzymes which are important in responses to hypoxia and chromatic modifications. In particular, α-KG is required for demethylases controlling chromatin modifications and DNA methylation, which exert effects on cell fate [22]. High intracellular concentrations of α-KG impact the histone and DNA methylation status of the embryonic stem cells to maintain their self-renewal and pluripotency. In vivo, primary GBM cell migration significantly increased after treatments with α-KG [20]. The PI3K/AKT/mTor pathway was also promoted by α-KG, suggesting that it may rely on an IDH1-α-KG axis. Lastly, α-KG can transaminate to glutamate, and increased levels of glutamate may promote tumor progression and invasion in glioma [23,24]. Conversely, in another study, overexpression of IDH1 in glioma IDH-wildtype did not cause changes in the cell cycle, apoptosis, and invasion ability; however, it did result in chemotherapy resistance to TMZ in vivo and in vitro [25]. Thus, improving our knowledge around IDH1 biology could be important.

Therapeutic advances to exploit metabolic vulnerabilities via Poly(ADP-ribose) glycohydrolase (PARG), which catalyzes the oligomerization of the essential cofactor NAD+, are being explored in IDH-mutant cancers. In glioma, PARG inhibition together with TMZ depletes NAD+ and leads to IDH-mutant cell death [18]. Strategies to target metabolic vulnerabilities are even more urgently required in IDH-wildtype GBM. Improving knowledge around IDH biology and mechanistic links with other genes is an area worthy of study as it could assist with the identification of new therapeutic targets. In vivo studies have demonstrated that aerobic glycolysis is insufficient to contribute to cellular anaplerosis and support GBM tumor cell growth. Therefore, GBM may selectively induce IDH1 mRNA, protein, and enzymatic activity to support high-grade glioma cells with macromolecules for rapid expansion [19]. The α-KG produced could lead to epigenetic reprogramming, altering the expression of metabolic genes supporting tumor growth or tumor suppressors and oncogenes. To this end, this study aimed to investigate IDH1 expression patterns in GBM and to further identify genes whose expression is associated with this gene. The study harnesses the power of artificial intelligence (AI) and “big data” gathered by the Cancer Genome Atlas (TCGA) as a large assembly of molecular profiles for glioma [8]. AI can recognize complex patterns in empirical data in a short amount of time and has previously been successfully applied to large cancer datasets for biomarker discovery [26,27]. Here, we utilize Atlas Correlation Explorer (ACE) software, which implements AI-based evolutionary algorithms for pattern recognition in an exploratory analysis of data [28]. Evolutionary algorithms apply Darwin’s evolutionary theory of natural selection for problem solving, quickly extracting new associations from big data. Genes identified as correlated with IDH1 expression were further explored for their biology and their utility as diagnostic and/or prognostic biomarkers. Biomarkers have a key role in drug development and in personalized medicine for patient diagnosis, outcome prediction for therapies, and informing about disease progression. Results expand our understanding of glioma etiology associated with IDH1 and altered cellular metabolism, identifying new mechanistic links and vulnerabilities, which could be targeted in future clinical trials for glioma IDH-wildtype.

## 2. Materials and Methods

### 2.1. In Silico Exploration of IDH1 Gene Expression in GBM

IDH1 expression in GBM was investigated using the web-based bioinformatics tool GlioVis [29]. Overexpression of IDH1 has been previously demonstrated in silico and in vivo in GBM [10,19,20]. Nevertheless, we sought to confirm this trend and explore it further. Thus, IDH1 expression in GBM was compared to non-tumor tissue in five published expression datasets [30,31,32,33,34] (see Supplementary Materials). Furthermore, IDH1 expression data were examined to determine whether copy number variation and IDH mutation status could explain the observed patterns in GBM using the TCGA-GBM dataset (RNA-seq) [8]. Lastly, IDH1 expression was compared within the GBM tumor to examine if there were differences between the leading edge, infiltrating tumor, cellular tumor, microvascular proliferation, and pseudopalisading cells around necrosis. Tissue for the five different anatomic structural features was collected using laser microdissection and underwent RNA-seq as part of the Ivy Glioblastoma Atlas Project (Ivy GAP) [35]. Statistical comparisons were implemented using *t*-tests with *p*-values corrected for multiple hypothesis testing using the Bonferroni method.

### 2.2. Data Description and Biomarker Identification Using ACE Software

ACE was used to identify genes with expression that was highly correlated with the glioma-associated gene, IDH1. Public data from the TCGA-LGG-GBM cohort warehoused within ACE and labelled as “GBL” were analyzed [8]. Samples were filtered based on age and IDH status using IDH1/2 mutation data. Only adults (>20 years) with IDH-wildtype gliomas, that have a worse overall prognosis, were included. Clinical information for “Cancer Type” and “Sample Type” were used to define three separate analyses: (1) All gliomas, (2) primary GBM, and (3) recurrent GBM (Table S1). An overview of the glioma subtypes based on the 2016 WHO classification system that were analyzed is provided in Table 1. The majority of tumors were primary, while only 14 were recurrent tumors. TCGA data for gene expression from the mRNASeq_RSEM_genes_normalized pipeline was analyzed. All genes were selected as the source measure, and IDH1 as the target. The ACE evolutionary algorithm analyzes an initial population of random “organisms” of source and target measures. Several data transformations (natural logarithm, arcsine, square root) are tested on each measure for their linear regression calculation. The fittest “individuals” are then selected based on their linear regression (R-squared) and used as the offspring for the next generation of the model. Additionally, random mutations are carried out on each organism and if the mutant organism has a higher fitness score than the original organism, it replaces it in the leaderboard. This process continues for many cycles, until the leaderboard of top-hits remains mostly consistent. By such time, the evolutionary algorithm has achieved 100% coverage, which means it has tested every data permutation at least twice. ACE was run for 12 hours per analysis. This ensured that it achieved 100% coverage and continued analysis for several hours thereafter. The leaderboard of top-hit genes, including their linear regression outputs (R-squared, line intercept, line slope) was saved. Correlations with a higher R-squared indicate a stronger association between target and source measures. The line slope, depending on its sign, distinguishes between negative or positive correlations between the source and the target. Results for top-hits were compared between analyses using Venny 2.1 [36]. The ACE algorithm, like real-world evolution, is based on randomness at every analytical stage (generation of new individuals, mating, and mutation). Given that each stage is random, consistent results between repeated analyses cannot be expected. Nevertheless, each ACE analysis was repeated and the overlap in top-hit gene lists was examined using Venny 2.1 [36].

### 2.3. Investigation of Genes as Potential Biomarkers and Validation Using GlioVis

The top-hit genes were investigated for their potential as diagnostic and/or prognostic biomarkers in primary and recurrent GBM using the web-based bioinformatics tool GlioVis [29]. Firstly, the mRNA expression of each gene was compared between non-tumor and GBM tissue samples using the TCGA-GBM dataset (RNA-seq). Next, mRNA expression was compared between different glioma subtypes according to the 2016 WHO classification system (oligodendroglioma, oligoastrocytoma, astrocytoma, GBM) using the TCGA LGG-GBM dataset (RNA-seq). For both analyses, pairwise t-tests were applied to compare mRNA expression between group levels with corrections for multiple hypothesis testing using the Bonferroni method. Potential diagnostic biomarkers were identified as having non-overlapping mRNA expression data points between GBM and non-tumor. A survival analysis was implemented to determine the prognostic potential of top-hit genes in primary and recurrent GBM (IDH-wildtype). High vs. low mRNA expression was compared in a Kaplan–Meier curve, using a median split and data from the TCGA-GBM (RNA-seq/Agilent 4502A). Results of both the Log-rank and Wilcoxon tests were examined for evidence of statistical significance (*p*-value < 0.05). The GBM datasets analyzed were limited to IDH-wildtype status and primary or recurrent depending on which analysis the gene was identified from.

For those genes identified as prognostic in TCGA-GBM, subsequent survival analyses were carried out to validate the biomarkers in five additional GBM cohorts in GlioVis [30,31,34,37,38].

### 2.4. Investigation of Gene Pathways Using DAVID Bioinformatic Resources

Database for Annotation, Visualization, and Integrated Discovery (DAVID) is a bioinformatics web-based tool for gene-enrichment and functional annotation analysis (GEFA) [39]. Analyses were carried out on the gene lists obtained from each analysis (All, GBM NR, GBM R) to measure gene enrichment in annotation terms using DAVID 6.8. Entrez accession numbers were used as the identifiers for genes. GEFA was implemented for KEGG pathways using a modified Fisher exact *p*-value (EASE score) with the default value of 0.1 for significance. The *p*-values adjusted for multiple hypothesis testing using the Bonferroni method were also estimated. All KEGG pathways associated with the genes in each analysis were also noted from the functional annotation tables.

### 2.5. Further Investigation of the Potential Biomarkers for Gene Alterations with cBioPortal 

The genes identified as potential biomarkers were further investigated for any genetic alterations that might explain their aberrant expression patterns in GBM compared to non-tumor tissue. Using cBioPortal [40], an oncoprint was plotted to explore the frequencies and types of gene mutations, amplifications, and deletions in the genes using the TCGA Glioblastoma (PanCancer Atlas) dataset (N = 378).

### 2.6. Further Investigations for Correlations between IDH1 mRNA Expression and Methylation, and with Protein Expression in GBM Using GlioVis

Using ACE, IDH1 mRNA expression (RSEM normalized) was compared against methylation by mean data from TCGA. Methylation by mean is estimated as the mean detection level of CpG methylation probes across a gene. For each potential biomarker gene, a plot including the linear regression and R-squared was examined to determine if there was a correlation. In addition, correlations between IDH1 mRNA expression (RNA-seq) and protein expression in GBM were further examined using GlioVis [29]. Reverse-phase protein array (RPPA) data from TCGA-GBM were analyzed by comparing two patient groups split based on the mRNA expression (Log2) data for the specified gene of interest, IDH1. The average protein expression of the high versus the low mRNA IDH1 expression groups was examined using the 50% and 75% quartile cut-offs and statistically compared using a t-test with *p*-values adjusted for multiple hypothesis testing using the Bonferroni method. Results for the gene’s proteins (*n* = 202) are ranked comparing the RPPA scores with high IDH1 mRNA expression, versus the low group.

## 3. Results

### 3.1. IDH1 Expression Patterns in GBM

IDH1 gene expression (mean ± stdev) was significantly overexpressed in adult primary GBM (11.52 ± 0.59) compared to non-tumor (8.85 ± 0.27) in the TCGA-GBM dataset (Figure S1a; *p*-value = 5.4 × 10^−16^), as previously reported [10,19]. This pattern of IDH1 overexpression in GBM compared to non-tumor was confirmed in five additional GBM datasets (*p*-values < 0.05), where fold increases of between 1.15 and 1.3 were observed (see Table S2). Overexpression of IDH1 in GBM did not appear to be due to copy number variation, as only 6.62% of GBM had gains (*n* = 10; Figure S1c). IDH1 expression was significantly higher in GBM IDH-wildtype (11.56 ± 0.58) compared to IDH-mutant (10.93 ± 0.43), now reclassified as grade IV astrocytoma IDH-mutant (Figure S1b, *p*-value = 2.6 × 10^−3^). Across the GBM tumor, IDH1 was differentially expressed, with higher levels observed in the cellular tumor (6.2 ± 0.69) and lower levels in the leading edge (4.85 ± 0.66; *p*-values < 0.05; Table S3). Equivalent levels of IDH1 gene expression were observed for infiltrating tumor (5.7 ± 0.7), microvascular proliferation (5.56 ± 0.39), and pseudopalisading cells around necrosis (5.56 ± 0.64; Figure S2).

### 3.2. Overview of the Gliomas Analyzed by ACE

In total, 668 glioma samples were analyzed (Table 1 and Table S1). According to the 2016 WHO classification system used at the time of initial diagnosis, most were primary tumors that were oligoastrocytoma (N = 16; 2.4%), anaplastic oligoastrocytoma (N = 10; 1.5%), oligodendroglioma (N = 10; 1.5%), astrocytoma (N = 9; 1.35%), anaplastic astrocytoma (N = 47; 7.04%), and glioblastoma (N = 562; 84.13%). A smaller number were recurrent tumors that included oligodendroglioma (N = 1; 0.15), astrocytoma (N = 1; 0.15), and glioblastoma (N = 12; 1.8%). Using the available TCGA mutation data to filter samples, all tumors were identified as IDH-wildtype. However, because a large amount of TCGA LGG-GBM data lacks mutation data for IDH1 (75.8%), it is likely that some mutant samples could not be excluded from the analysis. This number should have been small, however, given that only ~12.5% of GBMs are IDH1/2-mutant. These tumors are now reclassified as Grade IV Astrocytoma IDH-mutant according to the 2021 WHO classification system [2]. For comparative purposes only, we examined TCGA LGG-GBM clinical information for IDH status from their classifier. In total, 27 tumors were IDH-mutant, while 111 had an unknown status (Table 1). Thus, there was disagreement between our filtering using the TCGA mutation data and the classifier information in 20.65% of cases (N = 138).

### 3.3. Genes Associated with IDH1 in All Gliomas (IDH-Wildtype)

Analysis 1 included both primary and recurrent gliomas from Grades II to IV (Table 1 and Table S1). Of the 668 (85.9%) adult IDH-wildtype tumors analyzed, only 94 (14.1%) were not GBM. However, this may be an underestimate as some tumors may have been misdiagnosed/misclassified at the time due to a lack of molecular profiling information and/or the tumor being assessed on unrepresentative tissue. Nevertheless, the majority of adult gliomas that are of lower grades are IDH-mutant, so filtering using IDH1/2 mutation data reduced their number.

In total, 35 genes whose expression correlated with IDH1 expression were identified in analysis 1 (Table S4). Some 22 genes were downregulated and 13 upregulated in comparison to IDH1 expression. Top-hits for downregulated genes included TSPYL2 and KIAA1377, while BZW1 and RFC2 were the top-hits for upregulated genes. Analysis 1 was repeated and there was an overlap of 56% observed between leaderboards, with many of the top-hit genes such as TSPYL2, MINK1, EZH1, and JAKMIP1 identified again (results not shown). GEFA identified pyruvate metabolism as being overrepresented (Table 2). Nine of the other genes were associated with a range of pathways already established to be linked to cancer. These included the following KEGG pathways: cell cycle; Hippo signaling pathway; tight junction; HIF-1 signaling pathway; circadian rhythm; RNA transport, mRNA surveillance pathway, spliceosome; aminoacyl-tRNA biosynthesis, metabolic pathways; ErbB signaling pathway; and DNA replication, nucleotide excision repair, and mismatch repair (Table S7).

### 3.4. Genes Associated with IDH1 in Primary GBM (IDH-Wildtype)

Analysis 2 examined 562 primary non-recurrent GBMs (GBM NR). According to the TCGA clinical information for IDH status from a classifier, 76.15% (N = 428) were identified as IDH-wildtype (Table 1). A total of 35 genes were identified to be correlated with IDH1 expression in analysis 2 (Table S5). Some 26 genes were negatively correlated with IDH1, while 9 genes were positively correlated. Top-hits for downregulated genes included TSPYL2 and C20orf194, whilst PSMA3 and SNX6 were the top-hits for upregulated genes. There was an overlap of 75% between the genes in the leaderboard when analysis 2 was repeated (results not shown). GEFA identified three pathways as being overrepresented: ubiquitin-mediated proteolysis, focal adhesion, and mTOR signaling pathway (Table 2). Six of the other genes were associated with some of the following pathways: cytokine–cytokine receptor interaction; Jak-STAT signaling pathway; viral carcinogenesis; cholinergic synapse; proteasome; endocytosis; and microRNAs in cancer (Table S8).

### 3.5. Genes Associated with IDH1 in Recurrent GBM (IDH-Wildtype)

Analysis 3 examined 12 recurrent GBMs (GBM R), 9 of which were also confirmed as having IDH-wildtype status based on the classifier (75%; Table 1). A total of 34 genes were found to be associated with IDH1 (Table S6). Some 22 genes were negatively correlated and 12 genes were positively correlated with IDH1 expression. Top-hits for negatively correlated genes included MYH15 and C1orf198. Top-hits for positively correlated genes included TNFAIP6 and FKBP3. The oncogene PDGFA (subunit A of PDGF) was also found to be correlated with IDH1 in recurrent GBMs (Figure S3). There was little overlap between results of a repeated analysis (8.8%). Only three genes were identified in the leaderboard again (PLEKHM3, ULK1, TNFAIP6). GEFA revealed that the mTOR signaling pathway was also overrepresented by the gene list of GBM recurrent results, similar to GBM non-recurrent results (Table 2). Other pathways identified to be associated with the genes included other types of O-glycan biosynthesis, Notch signaling pathway, and signaling pathways regulating pluripotency of stem cells (PCGF3), for example (Table S9).

### 3.6. Further Analysis of Top-Hit Genes Associated with IDH1 as Potential Biomarkers

A total of 90 different genes were identified to be associated with IDH1 expression across the three ACE analyses. Comparison of the top-hits between all analyses revealed no genes common to all (Figure S4; Table 3). Ten genes were common between primary and recurrent GBM results. Only two genes were common between All and both the primary GBMs and the recurrent GBMs. Genes exclusive to the different analyses are also listed. For example, TSPYL2, PLEKHM3, and RFC2 were each identified in two analyses, while CIT and TMTC1 were exclusively identified when examining primary and recurrent GBMs, respectively (Table 3).

**Figure 1:**
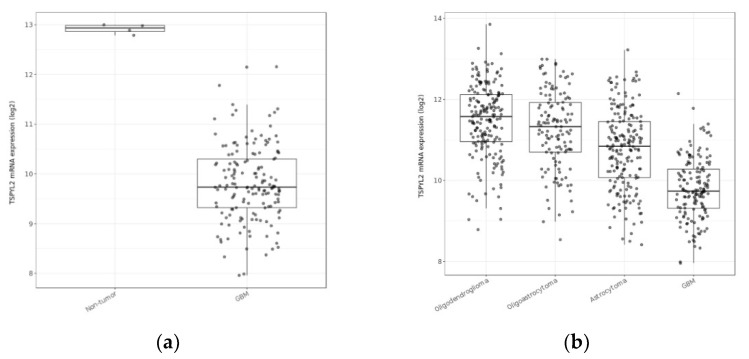

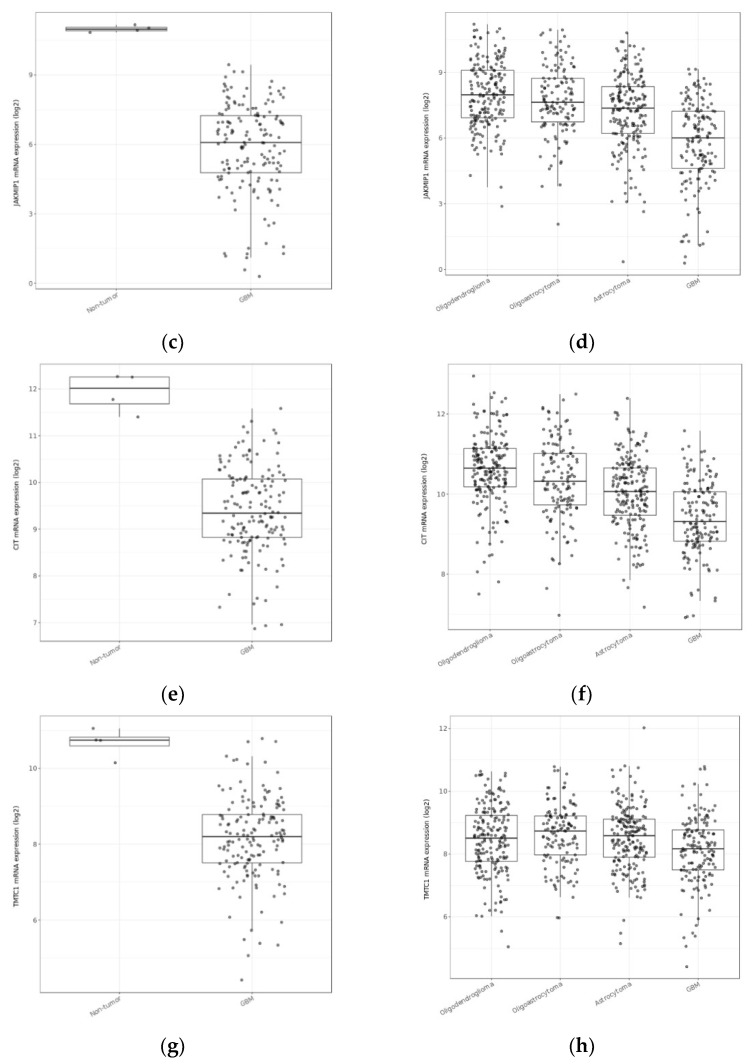
All pairwise comparisons of mRNA expression between GBM and non-tumor samples and also the other glioma subtypes were significantly different (*p* < 0.001; *t*-tests) for each of the potential prognostic genes: testis-specific protein Y-encoded 2; (**a**,**b**) (TSPYL2), Janus kinase and microtubule-interacting protein 1; (**c**,**d**) (JAKMIP1), citron rho-interacting serine/threonine kinase; (**e**,**f**) (CIT), and transmembrane O-mannosyltransferase targeting cadherins 1; (**g**,**h**) (TMTC1).

**Figure 2:**
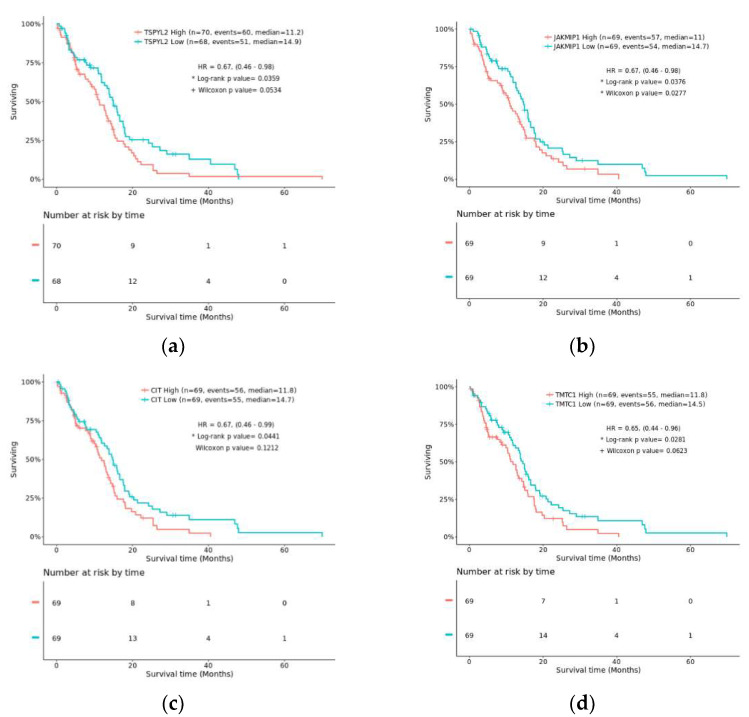
Results of the survival analysis with risk tables and Kaplan–Meier curves comparing overall survival of patients with high vs. low mRNA expression (median split). Each of the genes (**a**) TSPYL2; (**b**) JAKMIP1; (**c**) CIT; and (**d**) TMTC1 were prognostic for GBM (IDH-wildtype; *p* < 0.001; Log-rank test). TMTC1 was also prognostic for recurrent GBM (IDH-wildtype; *p* < 0.05; Log-rank test; not shown).

**Figure 3:**
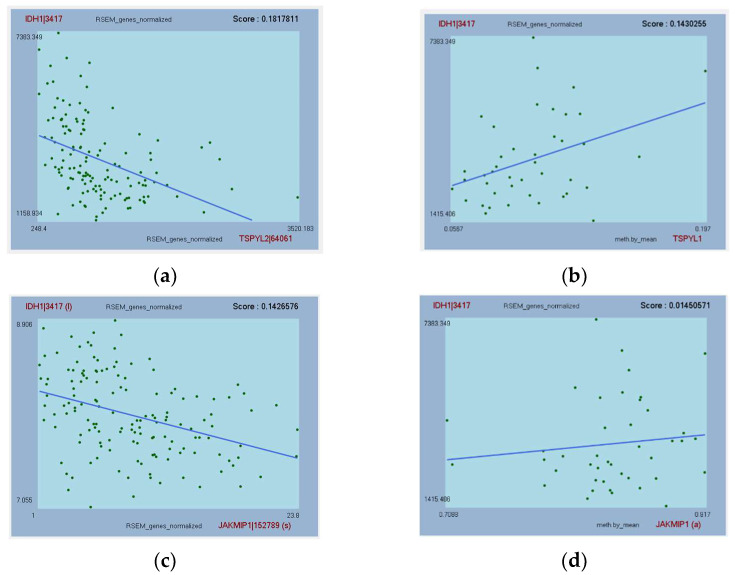

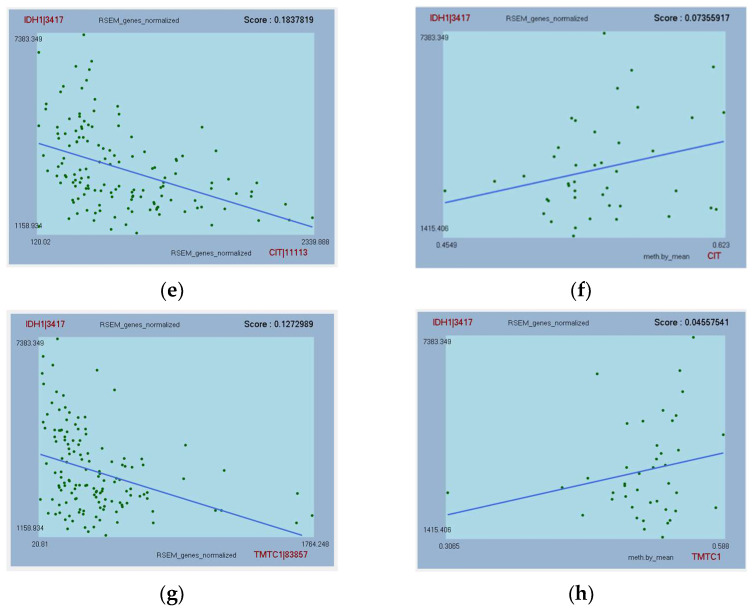
Correlations of IDH1 gene expression with expression and methylation data for TSPYL2 (**a**) and TSPYL1 * (**b**); JAKMIP1 (**c**,**d**); CIT (**e**,**f**); and TMTC1 (**g**,**h**). The linear regression line is provided as well as the R-squared “Score”. Parentheses indicate that transformed data (natural logarithm (l), arcsine (a), or square root (s)) provided a higher correlation in ACE. * Methylation data were not available for TSPYL2 in TCGA-GBM dataset, so TSPYL1 is presented instead.

Further investigations revealed four genes each as potential diagnostic and prognostic biomarkers (Figure 1, Figure 2 and Figure 3 and Figures S5 and S6). Potential diagnostic biomarkers MINK1, PLEKHM3, BZW1, and RCF2 had mRNA expression that differed significantly between GBM and the other glioma subtypes (*p*-values < 0.001; Figure S5). MINK1 and PLEKHM3 were significantly downregulated in GBM compared to non-tumor (*p*-values < 0.001; Figure S5B,C). The expression of these two genes was negatively correlated to IDH1 expression, while methylation was positively correlated (Figure S6B,C). BZW1 and RCF2 displayed the opposite trends; their expression was significantly upregulated in GBM compared to non-tumor (*p*-values < 0.001; Figure S5A,D). The expression of these two genes was positively correlated with IDH1 expression, while methylation was also positively correlated (Figure S6A,D).

Results of the survival analyses identified four potential prognostic biomarkers as TSPYL2, JAKMIP1, CIT and TMTC1. Kaplan–Meier curves revealed that the expression of these genes significantly affected the survival outcome of GBM (IDH-wildtype) patients (*p* < 0.05; Log-rank test; Figure 2) in the TCGA-GBM cohort. TMTC1 expression was also found to be potentially prognostic in recurrent GBM (IDH-wildtype) patients (*p* < 0.05; Log-rank test); however, sample sizes in comparative groups were below the minimum of ten required for a valid survival analysis.

The four genes identified as prognostic in the TCGA cohort were further tested in five independent GBM cohorts for validation (Table S10). Only TMTC1 was validated as prognostic in another GBM cohort. In five cases, the genes were not represented on the microarray so could not be tested.

TSPYL2, JAKMIP1, CIT, and TMTC1 were all significantly downregulated in GBM compared to non-tumor samples (*p* < 0.001; Figure 1). In addition, their expression in GBM was significantly different from the other glioma subtypes (*p* < 0.05; Figure 1). IDH1 expression was negatively correlated to TSPYL2, JAKMIP1, CIT, and TMTC1 expression, and positively correlated with their methylation for those genes tested (Figure 3).

Seven of the eight potential diagnostic and prognostic biomarkers were only affected by low frequencies of genetic alterations in GBM (>4%; Figure S7). Four were impacted by gene amplifications, while seven were impacted by missense mutations.

### 3.7. Genes Whose Protein Expression Is Associated with IDH1 Gene Expression

No protein results overlapped with the gene results from the ACE analysis. In addition to the methods differing fundamentally, the GBM cohort tested in the protein analysis was not IDH-wildtype-specific and only ~1% of genes/proteins were tested compared to the ACE analysis (*n* = 202). Thus, there were technical differences. Nevertheless, the top ten proteins that correlated with IDH1 mRNA expression in order were EGFR, Bax, S6, Chk2_pT68, eEF2K, GATA6, N.Cadherin, Stathmin, Rictor_pT1135, and Cyclin_D1 (Table S11). The top two proteins, EGFR and Bax, were significantly correlated with IDH1 mRNA expression (*p*-value adjusted < 0.05) when examining a 75% quartile cut-off between groups. Also listed within the top 33 proteins were beta.Catenin which may be associated with JAKMIP1, and P.Cadherin and E.Cadherin, which may be associated with TMTC1 (see Section 4).

## 4. Discussion

Brain tumor patients with GBM have a short survival rate; treatments are non-curative and consequently, tumors almost always recur. New therapeutic options are therefore urgently needed. Overexpression of IDH1 has been linked to disease progression in primary GBMs IDH-wildtype in vivo [19]. This study provides additional in silico evidence of IDH overexpression in GBM, which did not appear to be related to copy number gains and was comparatively higher in GBM IDH-wildtype compared to Grade IV Astrocytoma IDH-mutant. It was also interesting to note that IDH1 was differentially expressed across regions of the GBM tumor. Higher levels were observed in the cellular tumor and lower levels in the leading edge, which is involved in migration and invasion. In this study, a novel AI approach was applied to TCGA data to elucidate etiology associated with IDH1 and advance biomarker discovery in GBM. Expression measures for over 20,000 genes/transcripts from 668 glioma IDH-wildtype tumors were analyzed in an exhaustive search using evolutionary algorithms. ACE software identified the strongest correlations (positive/negative) in expression between IDH1 and all other genes within this large dataset. By applying Darwin’s evolutionary theory of natural selection for problem solving, results for three analyses using IDH-wildtype subsets (All gliomas, Primary GBM, Recurrent GBM) were obtained in a relatively short amount of time. In all, 90 genes that correlated with IDH1 expression were identified and those common or exclusive to the different subsets are reported. Genes that show potential as informative biomarkers and were common between analyses could potentially be used in diagnostic tests for early detection and as general targets for therapies. Genes exclusive to particular disease stages could be specific targets for therapies. All genes were further explored for their biology. Only nine genes were enriched in four core pathways, despite thirty-one genes being associated with KEGG pathways (see Supplementary Materials). Of these, only one gene (PDGFA) was a member of the known pathways involved in glioma disease development, according to DAVID (Figure S3). Thus, the majority of genes identified as being correlated with IDH1 expression were not associated with particular KEGG pathways.

Genes associated with IDH1 in both primary and recurrent GBMs IDH-wildtype were enriched for mTOR signaling (PDPK1, ULK1). In GBM, IDH1 overexpression leads to increased α-KG and primary GBM cell migration in vivo, which then promotes the PI3K/AKT/mTOR pathway [20]. Glycolytic reprogramming and GBM progression via PDPK1-dependent activation of PI3K/AKT/mTOR pathway is regulated by the transcription factor POU class homeobox 2 (POU2F2) [41]. The second gene highlighted was Unc-51-like autophagy-activating kinase 1 (ULK1). ULK1 is involved in autophagy (i.e. cell degradation) induction and has been linked to the development of neurogenerative disease [42]. In glioma, autophagy induces TMZ resistance but phosphorylation of ULK1 by T-LAK cell-originated protein kinase (TOPK), an upstream oncokinase, reduced autophagy and increased sensitivity of glioma cells to TMZ [43]. Inhibition of ULK1 restored radiosensitivity in human IDH-mutant but not IDH-wildtype glioma [44].

Analysis of All glioma tumors highlighted two genes involved in pyruvate metabolism that were associated with IDH1; these were glyoxalase I (GLO1) and pyruvate carboxylase (PC). Overexpression of GLO1 in glioma cell lines was associated with tumor cell proliferation, migration, and invasion [45]. Inhibition of GLO1 in GBM cell lines increased DNA-AGEs, stimulated RAGE expression, and induced apoptosis [46]. GBM relies on PC for the glucose-dependent replenishment of the TCA cycle with intermediates, a process known as anaplerosis [47]. The tumor suppressor NDRG2 inhibits PC expression in IDH-mutant and suppresses glioma growth but this was not observed for IDH-wildtype [48]. Furthermore, NDRG2 induces the ubiquitination and degradation of PC under glutamine deficiency, and NDRG2 loss leads to increased PC and PC-dependent anaplerosis and glioma tumorigenesis [48]. In vivo studies have demonstrated that aerobic glycolysis is insufficient to contribute to cellular anaplerosis and support GBM tumor cell growth [19]. Therefore, GBM may selectively induce IDH1 mRNA, protein, and enzymatic activity to support high-grade glioma cells with macromolecules for rapid expansion via these genes and pyruvate metabolism. These genes may represent metabolic vulnerabilities for targeting to inhibit anaplerosis and consequently inhibit GBM tumor growth.

Genes identified for primary GBM were enriched for several focal adhesions (COL4A6, PPP1CA, PDPK1), which can involve signaling molecules as well as structural links between membrane receptors (integrins) and the actin cytoskeleton. PDPK1 was also identified as a gene enriched in the mTOR signaling pathway. In zebrafish, collagen type IV alpha 6 chain (COL4A6) controls axon formation in glutamergic neurons (that produce glutamate) in the cerebellum by establishing and maintaining the integrity of the basement membrane [49]. In humans, perhaps a similar process occurs, influenced by IDH1 overexpression and increased α-KG levels; however, this would need to be experimentally investigated. Protein phosphatase 1 catalytic subunit alpha (PPP1CA) is a cell cycle regulator in the P53 network. In neuroblastoma cells, PPP1CA was repressed by miR-125b while its antisense RNA derepressed PPP1CA expression in human neural progenitor cells [50]. NF-κB pathway is constitutively activated by miR-125b, which in turn confers TMZ resistance in GBM [51].

For primary GBM, genes identified were also enriched for ubiquitin-mediated proteolysis (FBXO4, UBE2F, UBE3B), which plays an important role in cellular processes via selective protein degradation. F-box protein 4 (FBXO4) is one of the four subunits that make up the ubiquitin protein ligase complex, while ubiquitin-conjugating enzyme E2 F (putative) (UBE2F) and ubiquitin protein ligase E3B (UBE3B) are enzymes involved in the transfer and acceptance of ubiquitin to the targeted substrate. In esophageal cancer, dysregulation of the FBXO4-cyclin D1-RB axis promotes glutamine addiction and highlights a therapeutic weakness for overcoming CDK4/6 inhibitor resistance [52]. Thus, in GBM there may be a link between FBXO4 and α-KG that contributes to the TCA via glutamine metabolism. Regardless, E3 ubiquitin ligases are involved in apoptosis, maintaining glioma stem cells, and are emerging as abundant and promising targets for therapeutic interventions in GBM [53]. Other genes correlated with IDH1 identified in the recurrent GBM include polycomb group ring finger 3 (PCGF3). This gene is involved in signaling pathways regulating stem cells. PCGF3/5 positively regulates transcriptional activity in embryonic stem cells impacting the pluripotency factor TEX10 and they are also necessary for proper mesodermal lineage differentiation [54]. As high concentration levels of α-KG serve to maintain self-renewal of embryonic stem cells [22], perhaps they also influence PCGF3 in IDH-wildtype glioma stem cells.

Amongst the 90 genes identified in the biomarker discovery, eight displayed significant differences in mRNA expression in GBM compared to non-tumor and other glioma subtypes. The mRNA expression levels of four genes (TSPYL2, JAKMIP1, CIT, and TMTC1) significantly affected GBM patient outcome in the TCGA, as revealed by survival analyses (Figure 2). Only TMTC1 could be validated as prognostic in another GBM dataset, despite testing all genes in five independent datasets. Each of these potential prognostic biomarkers was negatively correlated with IDH1 and significantly downregulated in GBM compared to non-tumor. The methylation of JAKMIP1, CIT, and TMTC1 was positively correlated to IDH1 expression, suggesting that they are epigenetically reprogrammed in GBM. Methylation data were not available for testing TSPYL2; however, methylation of TSPYL1 was also positively correlated with IDH1 expression and it is documented for TSPYL2 also (see later). Two diagnostic (MINK1, PLEKHM3) biomarkers showed similar trends in expression while a further two potential diagnostic biomarkers (BZW1, RCF2) showed the opposite trend. Adopting a precision medicine approach, these genes may prove useful for patient stratification for determining treatment options. Given that the prognostic genes have aberrantly downregulated mRNA expression that negatively impacts patient survival, targeting these gene’s core pathways to revert mRNA back to normal levels perhaps could be a future treatment strategy.

Testis-specific protein Y-encoded 2 (TSPYL2) was the top-hit gene most negatively correlated with IDH1 expression in the All gliomas and primary GBM analyses. Survival analysis indicated that TSPYL2 is prognostic in GBM (IDH-wildtype). The TSPYL family is made up of nucleosome assembly proteins that are linked to several neurodevelopmental disorders [55]. TSPYL2 is recruited to promoters of specific EZH2 target genes in neurons, and enhances their expression for proper neuronal maturation and function [56]. When DNA is damaged, TSPYL2 plays an important role in inhibiting cell proliferation by stimulating P53 acetylation and P53-dependent cell death [57]. In gliomas, some members including TSPYL2 are downregulated due to epigenetic silencing and inhibit tumor growth [58]. Similarly, in the TCGA data, TSPYL2 mRNA expression was significantly downregulated in GBM compared to non-tumor. TSPYL2 is an essential component of the REST complex which is a tumor suppressor regulated by TGF-β signaling, which in turn can induce proliferative cell arrest [59]. Thus, downregulation of TSPYL2 may cause resistance to proliferative arrest in GBM tumors. TSPYL2 could potentially be an important target for therapies to slow the growth of GBM tumors.

Janus kinase and microtubule-interacting protein 1; marlin-1 (JAKMIP1) was identified as being associated with IDH1 expression in the All gliomas analysis. This microtubule-associated protein is normally highly expressed in the brain and is involved in the neuronal cytoskeleton [60]. However, in GBM, JAKMIP1 is downregulated, which can lead to abnormal formation of the brain cortex [61]. Dysregulation of microtubule-associated proteins such as JAKMIP1 is associated with neurodevelopmental diseases (see Lasser et al. for review) [62]. In lung cancer, JAKMIP1 overexpression has been implicated in cell proliferation through activating the Wnt/β-catenin pathway [63]. β-catenin was amongst the top proteins correlated with IDH1 expression. Further studies are needed to investigate the association of JAKMIP1 and IDH1 in GBM.

Citron rho-interacting serine/threonine kinase; CIT-K (CIT) was identified as being associated with IDH1 expression in the primary GBM analysis. CIT was downregulated in GBM and is prognostic for survival. CIT is involved in cell division to promote efficient cytokinesis. Silencing of CIT with microRNAs inhibits growth of medulloblastoma cells and induces cytokinesis failure, leaving the tumor cells more susceptible to chemotherapy [64]. CIT may be a promising target for a new therapy to slow GBM tumor growth. Experiments involving CIT gene silencing in GBM cell lines and in gene-knockout mice could be carried out to understand what effect the gene has on brain tumor growth or other.

Lastly, TMTC1 was identified as being associated with IDH1 expression in the recurrent GBM analysis. TMTC1 was downregulated in GBM and is prognostic for survival. TMTCs are enzymatically active sugar transferases belonging to the GT-C/PMT superfamily [65]. They are involved in the post-translational modification of cadherins, with O-linked mannose glycans, a process known as O-mannosylation [66]. Dysregulation of the tumor suppressor E-cadherin is an early molecular event in cancer and the interplay between O-mannosylation and N-glycosylation is a new mechanism responsible [67]. Genetic variants of TMTC1 have been linked to brain disorders such as schizophrenia, and their altered glycosylation may contribute to disease development [68]. Similarly, mutations in TMTC3 can cause severe brain malformation that affects neurons and glial cells [69]. The murine homolog of TMTC3, mSMILE, results in altering of the TGF-β signaling in embryonic fibroblasts [70]. TMTC3 acts as a binding partner for protein disulfide isomerase family A member 3 (PDIA3) and this gene’s expression plays a role in GBM-mediated pro-tumor activation of microglia [71]. P.Cadherin and E.Cadherin, which may be associated with TMTC1, were amongst the top proteins correlated with IDH1 expression. TMTC1 and the O-mannosylation pathway could be further explored to understand whether this pathway is involved in gliomagenesis.

Results of the analysis presented here identified basic leucine zipper and W2 domains 1 (BZW1), misshapen-like kinase 1 (MINK1), pleckstrin homology domain containing M3 (PLEKHM3), and replication factor C subunit 2 (RFC2) as potential diagnostic biomarkers in GBM. These genes may be useful for non-invasive liquid-biopsy testing for GBM. RFC2 to our knowledge has not been previously associated with glioma. This gene is involved in DNA replication and mismatch repair and is thought to have a role in the proliferation and metastasis of cancer cells [72]. In this study, PLEKHM3 was significantly downregulated in GBM compared to non-tumor, and its expression and methylation were negatively and positively correlated, respectively, with IDH1 expression. PLEKHM3 may act as a scaffold protein for AKT1. In ovarian cancer, PLEKHM3 is similarly downregulated, where it forms circular RNAs (circ-PLEKHM3) that regulate gene expression (similar to microRNAs) and act as a tumor suppressor [73]. Previously in GBM, the pleckstrin homology domain interacting protein (PHIP) was localized in the tumor leading edge and was identified as a key driver of migration, invasion, and angiogenesis [74]. Here, a comparison of gene expression between PLEKHM3 and IDH1 across the GBM tumor revealed the opposite trends, whereby PLEKHM3 was upregulated in the leading edge and downregulated in the cellular tumor (see Supplementary Materials). Potentially, PLEKHM3 may have a similar tumor suppressor role in GBM that is influenced by IDH1 or α-ketoglutarate; however, this would need to be experimentally investigated. From a translational perspective, experiments have shown that curcumin (turmeric’s active component) can restrain proliferation and facilitate apoptosis in ovarian cancer by regulating the circ-PLEKHM3/miR-320a/SMG21 axis [75]. Curcumin has previously been suggested to have a potential therapeutic role in GBM; however, no delivery system exists for testing it on brain tumors.

Lastly, it was interesting to note that EGFR protein levels correlated most strongly with IDH1 mRNA expression in GBM. EGFR is often genetically altered (amplified, mutated) in GBM. Whilst pre-clinical tests of EGRF inhibitors against GBM were promising, they lacked efficacy in GBM clinical trials but were effective in lung cancer trials. Perhaps their utility in combinatorial therapies targeting both the EGFR and STAT3 signaling pathways may hold better therapeutic promise for GBM [76]. The approach taken in this study has a number of limitations. The analysis of recurrent GBM IDH-wildtype tumors herein involved 12 samples, as TCGA data only have a very small proportion of recurrent GBM samples (2.1%). Thus, larger datasets are needed for recurrent GBM in order to facilitate translational discoveries. Results obtained here for recurrent GBM should be confirmed in a larger cohort. Lastly, in the GEFA analysis, only half of the genes analyzed were associated with a KEGG pathway. Those genes identified which have not been functionally characterized may be of interest for further GBM studies.

In conclusion, this study highlights a novel pipeline for biomarker discovery implementing AI-based evolutionary algorithms. Results provide new information on IDH-wildtype glioma etiology. The potential prognostic and diagnostic markers identified should be explored pre-clinically as targets for GBM therapies. Amongst these, CIT and TMTC1 have not been previously proposed for clinical utility. In addition, the mTOR signaling pathway was highlighted in this study. mTOR inhibitors are used as a maintenance treatment to control subependymal giant cell astrocytoma (SEGA) tumors in tuberous sclerosis [77]. Given that these drugs are in clinical use in this patient cohort, future pre-clinical studies could try to unlock these potential mechanisms of vulnerability in GBM patients to enable their use. Testing of inhibitors against the identified biomarker genes could be achieved using pre-clinical tumor models for glioma as in vivo and in vitro experiments [78]. For example, patient-derived GBM cell lines can be grown as 3D neurospheres and these can be tested in assays to determine if the gene inhibitors can reduce tumor cellular proliferation and facilitate apoptosis. Similarly, experimentation using pre-clinical animal models such as murine xenograft models can be established using patient-derived GBM tumors and tested to determine whether the inhibitors can reduce tumor growth and prolong overall survival of the pre-clinical models. These models may provide limited insight compared to patient-derived cancer organoid models (PDOs), which can more closely recapitulate the parental tumor tissue. In future, PDOs will be more routinely utilized for testing for precision oncology [79]. All assays could also be carried out with radiation together with radiosensitizers and/or other combinations of chemotherapies such as TMZ that are already used in the standard-of-care of brain tumors.

## Figures and Tables

**Table 1:** Overview of the Grade II–IV gliomas (IDH-wildtype) analyzed in this study. The subtypes listed are from the 2016 WHO classification system used at the time of initial diagnosis recorded by TCGA. In this study, IDH status was determined using TCGA mutation data for IDH1/2. Also listed, for comparative purposes only, is the clinical information for IDH status determined using the TCGA classifier approach (see Supplementary Materials of Ceccarelli et al., 2016 [11]).

					IDH Status (Classifier)		
Cancer Type	Primary/Recurrent	Grade	N	%	Wildtype	Mutant	Unknown
Oligoastrocytoma	Primary	II	16	2.40	16	0	0
Anaplastic Oligoastrocytoma	Primary	III	10	1.50	10	0	0
Oligodendroglioma	Primary	II	10	1.50	10	1	0
Oligodendroglioma	Recurrent	II	1	0.15	1	0	0
Astrocytoma	Primary	II	9	1.35	9	0	0
Astrocytoma	Recurrent	II	1	0.15	1	0	0
Anaplastic Astrocytoma	Primary	III	47	7.04	47	0	0
Glioblastoma	Primary	IV	562	84.13	428	23	111
Glioblastoma	Recurrent	IV	12	1.80	9	3	0
	Total		668	100	531	27	111

**Table 2:** Results of the gene-enrichment and functional annotation analyses for All gliomas, GBM NR, and GBM R gene lists. The genes in KEGG pathways that were considered to be “enriched” were identified using a *p*-value (EASE score) cut-off of 0.1 for significance. The *p*-values adjusted for multiple hypothesis testing using the Bonferroni method are also provided. KEGG terms, the identifier for each pathway used by the KEGG database are listed from the functional annotation clustering report. Count is the number of genes involved in the enriched pathway.

Analysis	KEGG Term	KEGG Pathway	Count	Gene Name	Entrez Accession Numbers	*p*-Value (EASE Score)	*p*-Value Adjusted (Bonferroni)
1. All	hsa00620	Pyruvate metabolism	2	GLO1, PC	5091, 2739	0.057	0.65
2. GBM NR	hsa04120	Ubiquitin-mediated proteolysis	3	FBXO4, UBE2F, UBE3B	26,272, 140,739, 89,910	0.023	0.69
2. GBM NR	hsa04510	Focal adhesion	3	COL4A6, PPP1CA, PDPK1	1288, 5499, 5170	0.048	0.92
2. GBM NR	hsa04150	mTOR signaling pathway	2	PDPK1, ULK1	5170, 8408	0.097	0.99
3. GBM R	hsa04150	mTOR signaling pathway	2	PDPK1, ULK1	5170, 8408	0.089	0.99

**Table 3:** Comparisons of the gene lists between the different analyses (All, GBM NR, GBM R) identified genes common between analyses and those exclusive to each analysis. Genes identified as potential biomarkers after further analysis are highlighted in bold.

Genes Common Between:			Genes Exclusive To:		
All & GBM NR	All & GBM R	GBM NR & GBM R	All	GBM NR	GBM R
**TSPYL2** C9orf45	**RFC2** PPIA	C20orf194 MECP2 **PLEKHM3** C1orf198 MLLT6 HDAC5 ULK1 PDPK1 PSMA3 FKBP3	MYH7B **MINK1** EZH1 TOM1L2 GABPB1 NRG3 CRY2 GRAMD1 **JAKMIP1** PC C5orf53 PLCXD3 SYNE1 TCEAL3 FNBP1 TBRG1 LLGL2 C10orf28 QRSL1 ZNF224 BZW1 DBF4 MED20 ILF2 C6orf153 ZNF410 EIF4A3 TIMP1 ZNF277 GLO1	MPL LPAL2 FAM189A1 COL4A6 COLQ **CIT** UBE3B FBRS KCNQ5 PDZD8 FAM53C FAT3 IQCF1 MYOD1 DENND MFSD4 SNX6 C2orf80 NCRNA PPP1CA UBE2F FBXO4 ZEB1	MYH15 HSFX2 ANKRD24 ZDHHC11 **TMTC1** EDA FLYWCH1 TULP4 PER3 PCGF3 ABCC5 KIAA0355 MFNG FEZF1 TNFAIP6 PDGFA MEMO1 FAM3C COMMD1 FAM32A FAM98C FAM131B

## Data Availability

The TCGA LGG-GBM data used in this paper are publicly available from the Broad Institute Firehose https://gdac.broadinstitute.org/, accessed on 1 March 2022. They are also warehoused within ACE, labelled as “GBL”. ACE is free and publicly available software, which can be downloaded from GitHub (https://github.com/AlanRGilmore/ACE, accessed on 1 March 2022) and implemented as a Windows desktop application.

## Supplementary Materials

The following supporting information can be downloaded at: https://www.mdpi.com/article/10.3390/cimb44070206/s1. References [8,30,31,32,33,34,37,38] are cited in Supplementary Materials.
