# Supplementary material for: Using AI-Based Evolutionary Algorithms to Elucidate Adult Brain Tumor (Glioma) Etiology Associated with IDH1 for Therapeutic Target Identification
Source: Curr Issues Mol Biol. 2022 Jul 2;44(7):2982–3000. doi: 10.3390/cimb44070206 (PMC9323620; doi:10.3390/cimb44070206)
Supplement: Supplementary file 1 [file cimb-44-00206-s001.zip › cimb-1684327-supplementary.pdf]

Supplementary materials

Using AI-based evolutionary algorithms to elucidate adult brain tumor (glioma) etiology associated with IDH1 for therapeutic target identification

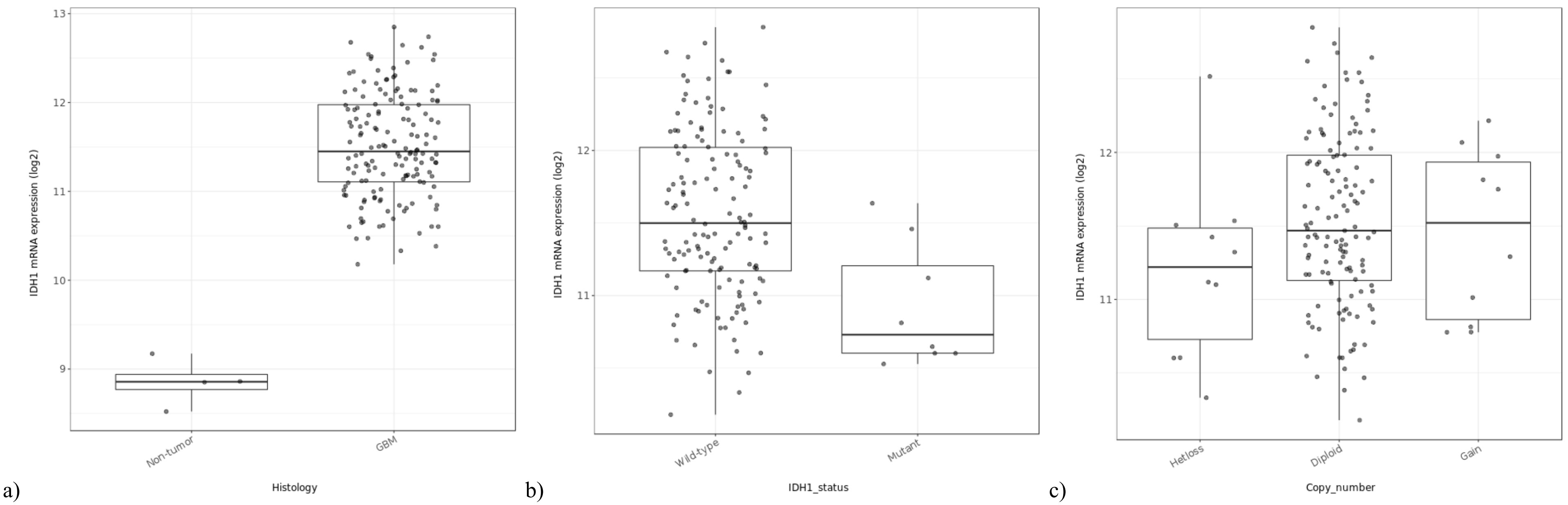

Figure S1. IDH1 gene expression was significantly overexpressed in adult Primary GBM compared to non-tumour (a; *P*-value= 5.4E-16) and between GBM IDH-wildtype and IDH-mutant (b, *P*-value= 2.6E-03), now re-classified as Grade IV Astrocytoma IDH-mutant. Overexpression of IDH1 did not appear to be due to copy number gains (c). The data analysed was the TCGA-GBM dataset (RNA-seq) and all *P*-values of the t-tests were corrected for multiple testing using the Bonferroni method.

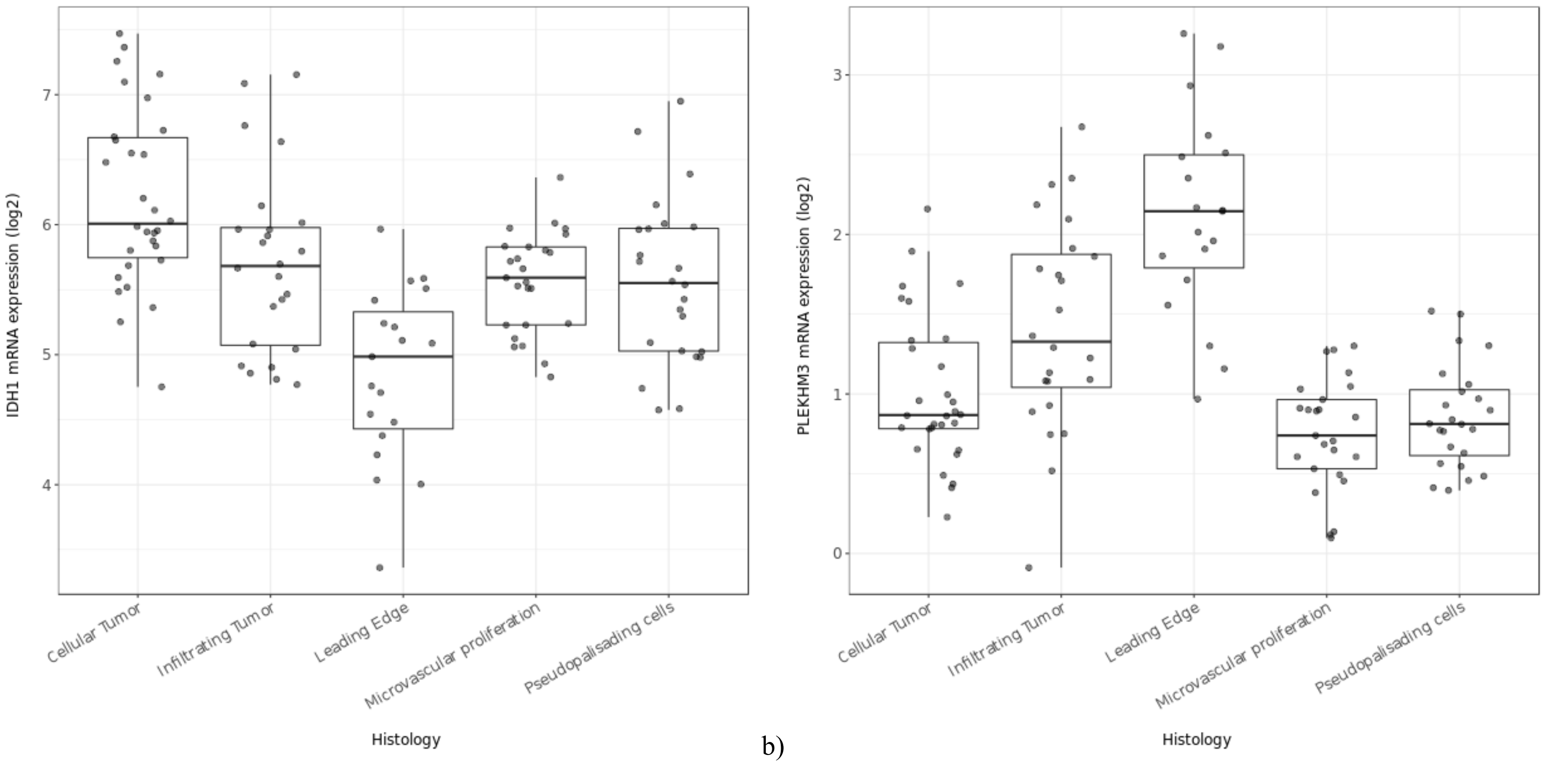

a) b)

Figure S2. Comparison of gene expression between different anatomic structural features in the GBM tumour (Leading Edge, Infiltrating Tumor, Cellular Tumor, Microvascular Proliferation, Pseudopalisading Cells Around Necrosis) for the genes IDH1 (a) and PLEKHM3 (b). The data analysed is the Ivy GAP dataset (RNA-seq)[35].

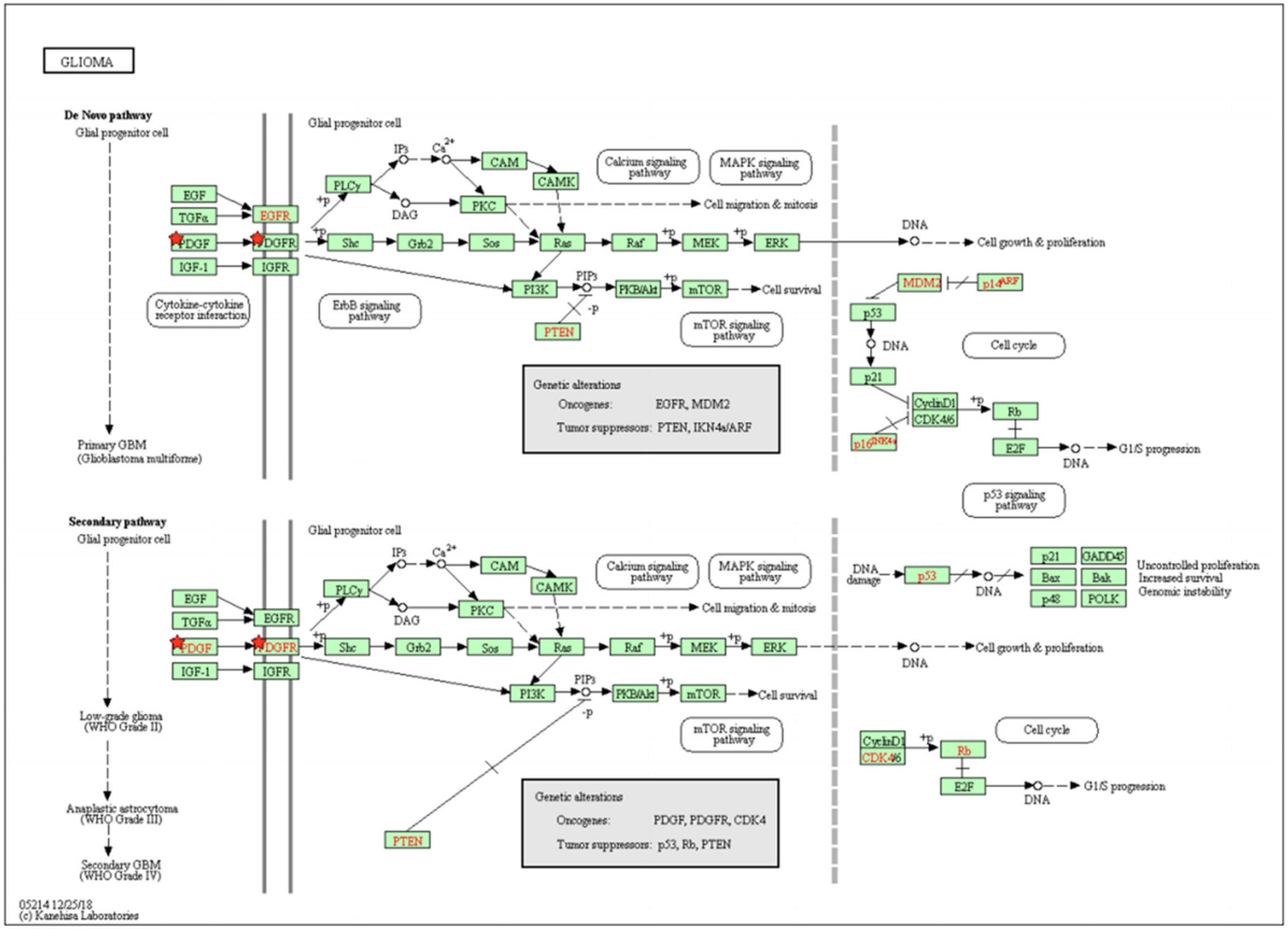

List genes are shown in red

Figure S3. Results from the GEFA analysis of recurrent GBMs identified only one gene, *PDGFA* (starred) from the genetic pathways known to be involved in glioma *de novo* and recurrent disease development as illustrated in the graphic provided by DAVID.

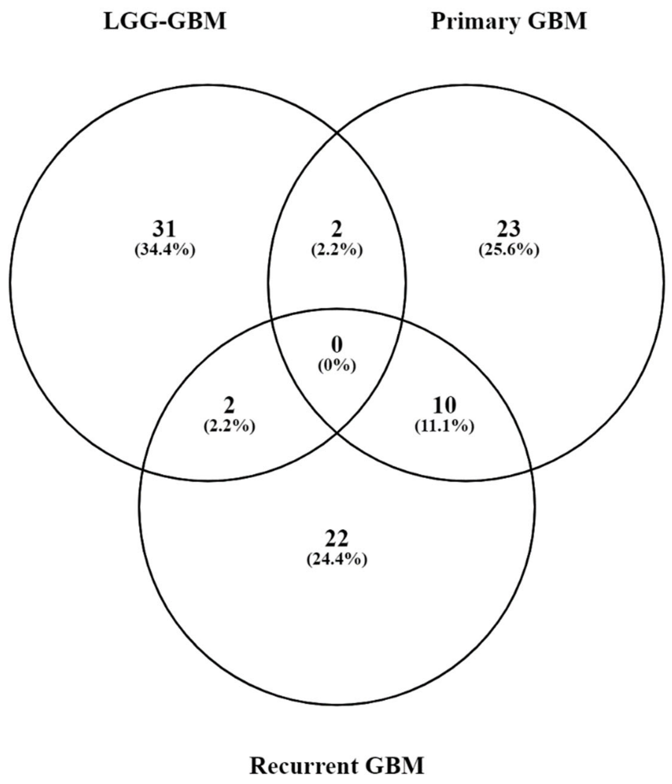

Figure S4. Comparison of the ACE results for analyses 1) All (LGG-GBM), 2) Primary GBM (GBM NR) and 3) Recurrent GBM (GBM R). The common genes are identified in the intersections overlap, while the unique genes are identified in the relative complements.

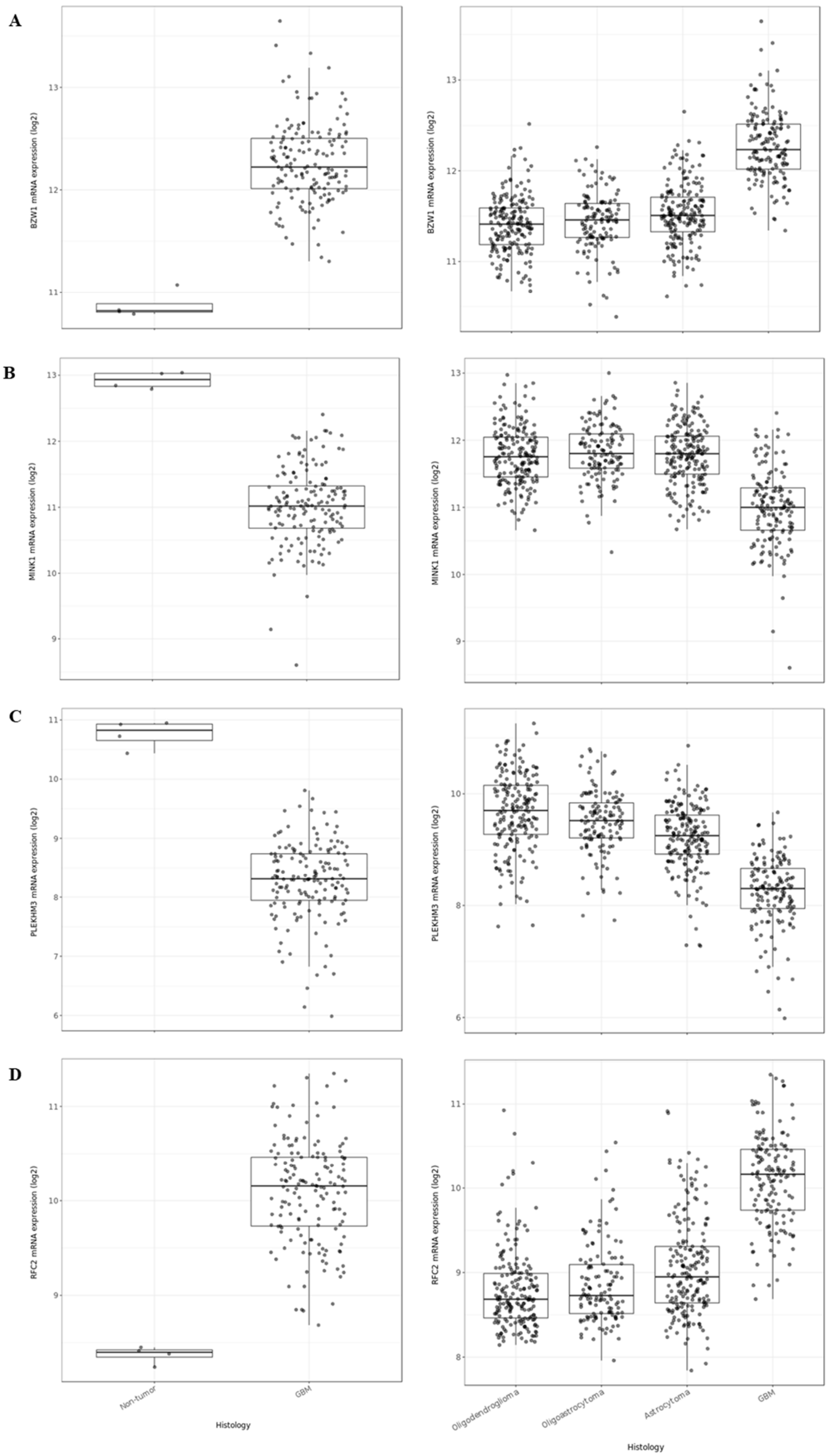

Figure S5. Comparisons of GBM mRNA expression to non-tumour tissue and to all other glioma subtypes were significantly different ($P < 0.001$; t-tests) for the potential diagnostic genes *BZW1* (Basic Leucine Zipper And W2 Domains 1; rows A), *MINK1* (Misshapen Like Kinase 1; B), *PLEKHM3* (Pleckstrin Homology Domain Containing M3; C) and *RFC2* (Replication Factor C Subunit 2; D).

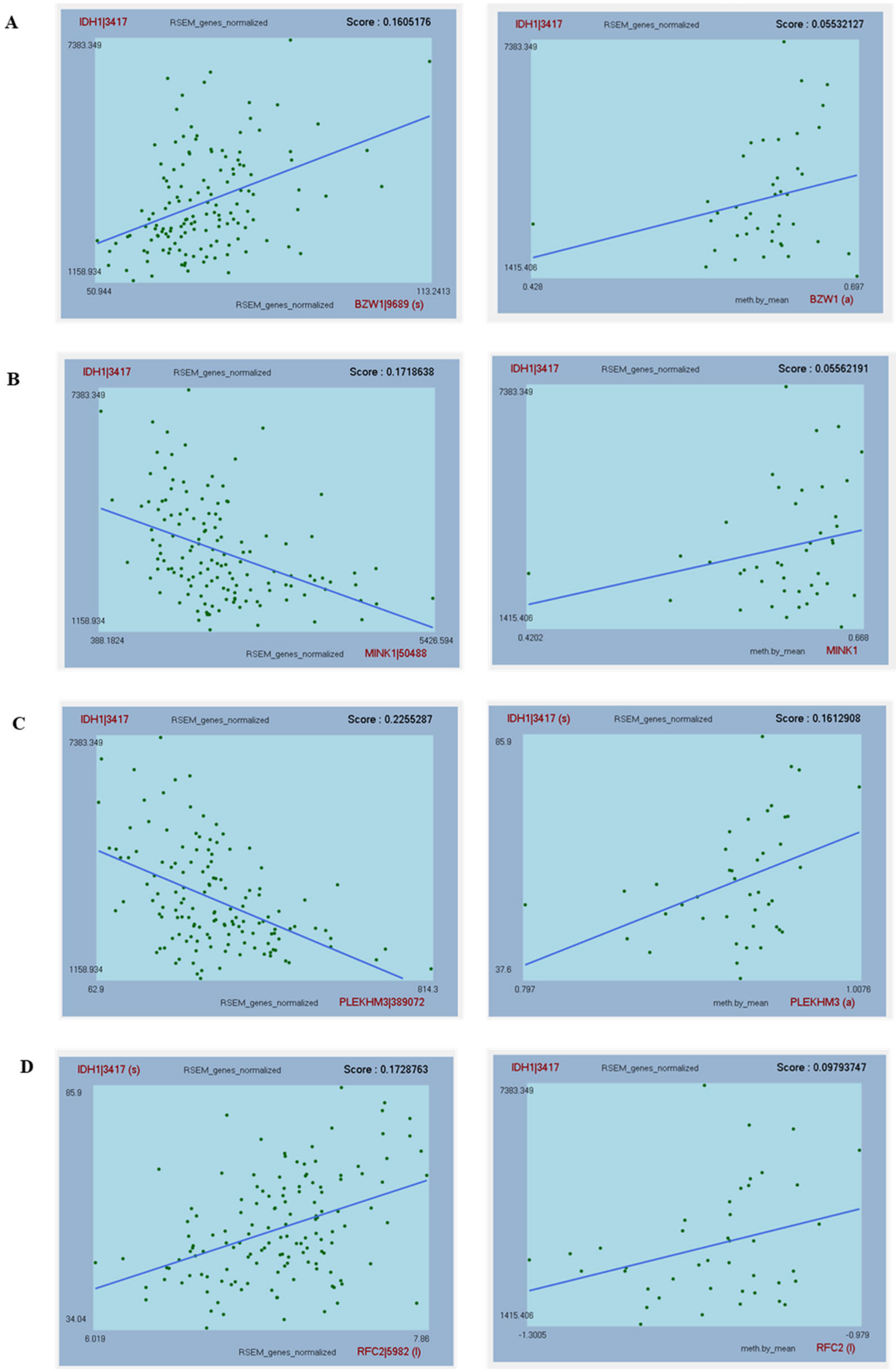

Figure S6. Correlations of IDH1 mRNA expression with methylation data for the potential diagnostic genes BZW1 (Basic Leucine Zipper And W2 Domains 1; rows A), MINK1 (Misshapen Like Kinase 1; B), PLEKHM3 (Pleckstrin Homology Domain Containing M3; C) and RFC2 (Replication Factor C Subunit 2; D) in GBM (IDH-wildtype). The linear regression trend line and the R-squared is provided as the "Score". Parentheses indicate which data transformation (natural logarithm (l), arcsine (a) or square root (s)) provided the highest correlation in ACE.

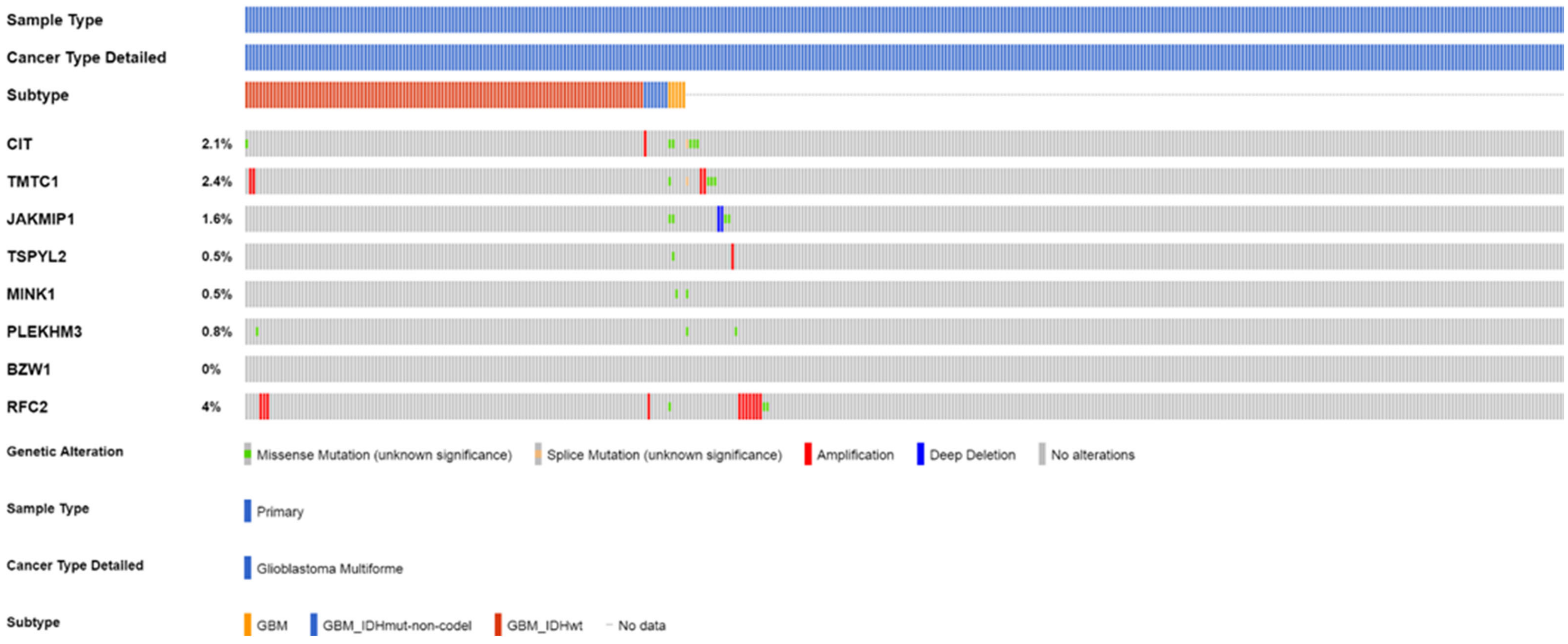

Figure S7. Oncoprint of the frequencies (%) and types of genetic alterations (missense / splice mutations, amplifications, deletions) detected in the potential biomarker genes in the TCGA Glioblastoma (PanCancer Atlas) dataset (N=378). Tumour subtype annotations, based on the 2016 WHO classification system used at the time of sample analysis, are provided for GBM IDH-wildtype individuals (red), GBM (yellow), GBM IDH-mutant non-codeleted (blue) and no data.

Table S1. Overview of the three separate analyses carried out on the TCGA LGG-GBM cohort using ACE. Samples were filtered based on *IDH1/2* mutation data and TCGA clinical information for age, cancer type and sample type.

**Filtering criteria:**	**Analysis 1-All gliomas**	**Analysis 2- GBM NR**	**Analysis 3- GBM R**
**Age (years)**	>20	>20	>20
**IDH 1/2 mutations**	IDH-wildtype	IDH-wildtype	IDH-wildtype
**Cancer Type**	All	GBM	GBM
**Sample Type**	All	Primary	Recurrent
**Number of samples**	668	562	12

Table S2. Comparison of IDH1 expression in GBM compared to non-tumor tissue in six independent GBM datasets.

**Dataset/Reference**	**GBM / GBM IDH-wildtype**	**Data platform**	**GBM Mean**	**Stdev**	**Non-Tumour Mean**	**Stdev**	**Fold increase**	***P*-value**
TCGA-GBM[8]	GBM	RNA-seq	11.52	(0.59)	8.85	(0.27)	1.30	5.40E-16
Gravendeel[30]	GBM IDH-wildtype	Microarray	10.93	(0.52)	8.77	(0.44)	1.25	1.1 E-22
Grzmil[33]	GBM	Microarray	8.19	(0.74)	6.85	(0.28)	1.20	2.60E-02
Kamoun[32]	GBM	Microarray	10.06	(0.94)	8.28	(0.19	1.21	2.80E-05
Rembrandt; Madhavan[31]	GBM	Microarray	8.87	(0.55)	7.73	(0.26)	1.15	1.70E-22
Murat[34]	GBM	Microarray	9.91	(0.76)	8.27	(0.60)	1.20	2.70E-06

Stdev = standard deviation

Table S3. Comparison of IDH expression between different anatomic structural features in the GBM tumor (Leading Edge, Infiltrating Tumor, Cellular Tumor, Microvascular Proliferation, Pseudopalisading Cells Around Necrosis). The data analysed is the Ivy GAP dataset (RNA-seq)[35]. *P*-values have been corrected for multiple hypothesis testing using the Bonferroni method.

	Cellular Tumor	Infiltrating Tumor	Leading Edge	Microvascular proliferation
Infiltrating Tumor	4.50E-02	-	-	-
Leading Edge	2.80E-10	2.00E-04	-	-
Microvascular proliferation	2.50E-03	NS	3.00E-03	-
Pseudopalisading cells	2.90E-03	NS	3.40E-03	NS

- = not applicable; NS = non-significant

Table S4. Results of Analysis 1 examining All gliomas IDH-wildtype. Genes positively and negatively correlated with *IDH1* mRNA expression are listed as well as their linear regression outputs (R-squared, Line Intercept, Line Slope). Potential biomarkers are highlighted in bold. Results of the expression comparison of GBM vs Non-tumour is provided (*P*-value; t-test).

No.	**Source Measure | Entrez accession number**	**Target Measure | Entrez accession number**	**R-Squared**	**Line Intercept**	**Line Slope**	***P*-value**
1	***TSPYL2|64061***	IDH1|3417	0.370293	11127.22	-1204.35	**1.90E-13**
2	*KIAA1377|57562*	IDH1|3417	0.364296	5812.218	-641.242	NA
3	*MYH7B|57644*	IDH1|3417	0.328003	3831.119	-178.132	**1.20E-07**
4	***MINK1|50488***	IDH1|3417	0.327042	4357.918	-0.57321	**9.00E-10**
5	*C9orf45|81571*	IDH1|3417	0.325283	7490.466	-939.717	NA
6	*EZH1|2145*	IDH1|3417	0.323603	4829.073	-2.12736	**1.20E-07**
7	*TOM1L2|146691*	IDH1|3417	0.322905	5707.141	-66.1072	**3.30E-09**
8	*NRG3|10718*	IDH1|3417	0.314884	5084.944	-536.706	**3.80E-06**
9	*CRY2|1408*	IDH1|3417	0.306198	3810.023	-1.08131	**5.10E-12**
10	*GRAMD1B|57476*	IDH1|3417	0.302988	6376.04	-778.192	**6.10E-09**
11	***JAKMIP1|152789***	IDH1|3417	0.302144	3721.77	-76.4849	**4.20E-07**
12	*PC|5091*	IDH1|3417	0.30086	4015.313	-1.09481	**7.40E-05**
13	*C5orf53|492311*	IDH1|3417	0.296354	3999.527	-1.87004	NA
14	*PLCXD3|345557*	IDH1|3417	0.265374	3568.113	-85.3524	**4.20E-07**
15	*SYNE1|23345*	IDH1|3417	0.199191	3416.291	-0.36239	**7.90E-09**
16	*TCEAL3|85012*	IDH1|3417	0.193251	4067.78	-1.24054	**2.20E-03**
17	*FNBP1|23048*	IDH1|3417	0.154721	4028.52	-0.75106	1.60E-01
18	*TBRG1|84897*	IDH1|3417	0.134961	4185.672	-1.46282	**3.00E-16**
19	*LLGL2|3993*	IDH1|3417	0.110425	3231.935	-4.57289	6.40E-01
20	*C10orf28|27291*	IDH1|3417	0.041739	3721.647	-5.46299	NA
21	*QRSL1|55278*	IDH1|3417	0.002626	2775.761	-0.04966	**2.30E-02**
22	*ZNF224|7767*	IDH1|3417	0.027819	3172.672	-2.51985	3.00E-01
23	***BZW1|9689***	IDH1|3417	0.367812	-18356.7	2522.228	**6.2E-11**
24	***RFC2|5982***	IDH1|3417	0.323548	1085.007	1.617025	**2.8E-09**
25	*GABPB1|2553*	IDH1|3417	0.317651	624.9755	6.077608	**8.6E-07**
26	*DBF4|10926*	IDH1|3417	0.31074	-3111.64	1059.284	**3.7E-09**
27	*MED20|9477*	IDH1|3417	0.307837	426.1052	4.777814	**3.2E-11**
28	*PPIA|5478*	IDH1|3417	0.300284	1434.563	0.18438	**1.7E-05**
29	*ILF2|3608*	IDH1|3417	0.304503	-13558	1980.796	**2.2E-07**
30	*C6orf153|88745*	IDH1|3417	0.301933	185.1627	3.723208	NA

31	*ZNF410|57862*	IDH1|3417	0.298686	1332.796	1.222343	**2.6E-05**
32	*EIF4A3|9775*	IDH1|3417	0.296284	236.0151	1.485654	**8.3E-16**
33	*TIMP1|7076*	IDH1|3417	0.174988	-1958.93	554.2538	**3.1E-05**
34	*ZNF277|11179*	IDH1|3417	0.167221	1000.353	5.136995	6.2E-02
35	*GLO1|2739*	IDH1|3417	0.137056	1488.493	0.410329	5.1E-02

Table S5. Results of Analysis 2 examining Primary GBMs IDH-wildtype. Genes positively and negatively correlated with *IDH1* mRNA expression are listed as well as their linear regression outputs (R-squared, Line Intercept, Line Slope). Potential biomarkers are highlighted in bold. Results of the expression comparison of GBM vs Non-tumour is provided (*P*-value; t-test).

No.	**Source Measure | Entrez accession number.**	**Target Measure | Entrez accession number.**	**R-Squared**	**Line Intercept**	**Line Slope**	**P-value**
1	***TSPYL2|64061***	IDH1|3417	0.264346	133.438	-11.5015	**1.90E-13**
2	*C20orf194|25943*	IDH1|3417	0.240031	82.45618	-0.98677	**2.90E-06**
3	*C9orf45|81571*	IDH1|3417	0.238047	89.28619	-7.08891	NA
4	*MECP2|4204*	IDH1|3417	0.225342	87.701	-0.90064	**6.0E-05**
5	***PLEKHM3|389072***	IDH1|3417	0.22512	68.80836	-0.04097	**4.1E-12**
6	*MPL|4352*	IDH1|3417	0.223203	72.49058	-6.23847	6.5E-02
7	*C1orf198|84886*	IDH1|3417	0.222773	64.03928	-0.00392	1.00E+00
8	*LPAL2|80350*	IDH1|3417	0.210146	60.21885	-1.64076	**5.6E-08**
9	*FAM189A1|23359*	IDH1|3417	0.209434	61.82635	-0.04344	**1.8E-06**
10	*MLLT6|4302*	IDH1|3417	0.208285	69.57452	-0.00641	**1.2E-03**
11	*COL4A6|1288*	IDH1|3417	0.201885	63.54987	-0.81898	**7.7E-03**
12	*COLQ|8292*	IDH1|3417	0.200059	81.87206	-6.24164	1.6E-01
13	*HDAC5|10014*	IDH1|3417	0.199897	72.26836	-0.0132	**6.5E-07**
14	*ULK1|8408*	IDH1|3417	0.199178	70.42609	-0.01608	**3.4E-10**
15	***CIT|11113***	IDH1|3417	0.198902	71.85674	-0.61311	**2.3E-07**
16	*PDPK1|5170*	IDH1|3417	0.198605	83.57336	-0.88322	**1.7E-08**

17	*UBE3B|89910*	IDH1|3417	0.196195	192.9497	-20.2029	**8.4E-03**
18	*FBRS|64319*	IDH1|3417	0.174197	72.76978	-0.01447	4.3E-01
19	*KCNQ5|56479*	IDH1|3417	0.133266	67.78359	-2.90183	**9.5E-06**
20	*PDZD8|118987*	IDH1|3417	0.120935	100.2102	-8.09001	**1.2E-03**
21	*FAM53C|51307*	IDH1|3417	0.085009	72.3286	-0.01277	**3.2E-04**
22	*FAT3|120114*	IDH1|3417	0.070018	59.17591	-0.00595	**3.2E-02**
23	*IQCF1|132141*	IDH1|3417	0.051652	56.88142	-2.7601	**2.9E-04**
24	*MYOD1|4654*	IDH1|3417	0.034716	56.28747	-1.47958	7.4E-01
25	*DENND4C|55667*	IDH1|3417	0.032928	68.18118	-0.47349	**2.7E-02**
26	*MFSD4|148808*	IDH1|3417	0.01578	56.91388	-0.00726	NA
27	*PSMA3|5684*	IDH1|3417	0.220142	41.85515	0.012581	**1.9E-03**
28	*SNX6|58533*	IDH1|3417	0.217857	40.90828	0.009709	**1.9E-05**
29	*C2orf80|389073*	IDH1|3417	0.209159	45.82205	0.681165	**2.5E-02**
30	*NCRNA00152|112597*	IDH1|3417	0.208598	16.42328	6.611343	NA
31	*FKBP3|2287*	IDH1|3417	0.208125	41.97928	0.0098	1.3E-01
32	*PPP1CA|5499*	IDH1|3417	0.20049	-64.03	14.9607	**1.5E-04**
33	*UBE2F|140739*	IDH1|3417	0.194277	-54.1266	16.31309	1.3E-02
34	*FBXO4|26272*	IDH1|3417	0.071608	49.57985	0.017523	**3.4E-08**
35	*ZEB1|6935*	IDH1|3417	0.030926	51.5971	0.001492	**3.2E-02**

Table S6. Results of Analysis 3 examining Recurrent GBMs IDH-wildtype. Genes positively and negatively correlated with IDH1 mRNA expression are listed as well as their linear regression outputs (R-squared, Line Intercept, Line Slope). Potential biomarkers are highlighted in bold. Results of the expression comparison of GBM vs Non-tumour is provided (*P*-value; t-test).

No.	**Source Measure | Entrez accession number**	**Target Measure | Entrez accession number**	**R-Squared**	**Line Intercept**	**Line Slope**	**P-value**
1	*MYH15|22989*	IDH1|3417	0.255739	4773.214	-566.555	**1.1E-03**
2	*C1orf198|84886*	IDH1|3417	0.239035	11402.55	-1090.56	1.0E+00
3	*MECP2|4204*	IDH1|3417	0.236831	16177.05	-1818.61	**6.0E-05**
4	*C20orf194|25943*	IDH1|3417	0.224401	4799.93	-2.02762	**2.9E-06**
5	***PLEKHM3|389072***	IDH1|3417	0.220246	4770.471	-4.78111	**4.1E-12**
6	*HSFX2|100130086*	IDH1|3417	0.217676	6218.176	-1033.7	**2.1E-05**
7	*MLLT6|4302*	IDH1|3417	0.203389	4846.312	-0.74397	**1.2E-03**
8	*PDPK1|5170*	IDH1|3417	0.201535	14010.73	-1565.87	**1.7E-08**
9	*ANKRD24|170961*	IDH1|3417	0.198757	6170.126	-682.469	**4.1E-08**
10	*ZDHHC11|79844*	IDH1|3417	0.197632	5279.06	-567.873	6.9E-02
11	*ULK1|8408*	IDH1|3417	0.197532	6750.358	-117.564	**3.4E-10**
12	***TMTC1|83857***	IDH1|3417	0.195743	7386.032	-739.658	**1.0E-05**
13	*EDA|1896*	IDH1|3417	0.192429	6003.033	-736.383	2.9E-01
14	*HDAC5|10014*	IDH1|3417	0.189314	5115.221	-1.50059	**6.5E-07**
15	*FLYWCH1|84256*	IDH1|3417	0.188069	15884.08	-1885.04	**2.4E-07**
16	*TULP4|56995*	IDH1|3417	0.187534	10126.53	-1126.03	**1.7E-06**
17	*PER3|8863*	IDH1|3417	0.098393	3775.779	-1.31944	**2.8E-03**
18	*PCGF3|10336*	IDH1|3417	0.069658	4110.637	-0.94167	2.8E-01
19	*ABCC5|10057*	IDH1|3417	0.037241	3742.702	-0.5788	**1.7E-03**
20	*KIAA0355|9710*	IDH1|3417	0.025365	3890.319	-0.82642	**1.9E-03**
21	*MFNG|4242*	IDH1|3417	2.41E-05	3304.079	-13.6665	**7.0E-09**
22	*FEZF1|389549*	IDH1|3417	0.015574	3283.845	-2.33471	2.9E-01
23	*TNFAIP6|7130*	IDH1|3417	0.257412	-571.38	690.3013	**5.4E-04**
24	*FKBP3|2287*	IDH1|3417	0.221723	1572.664	1.192386	1.3E-01
25	*PSMA3|5684*	IDH1|3417	0.209421	-118.923	102.9441	**1.9E-03**
26	*PDGFA|5154*	IDH1|3417	0.207366	2447.163	0.462412	1.6E-01

27	*MEMO1|51072*	IDH1|3417	0.199004	1586.797	4.063575	**7.0E-03**
28	*PPIA|5478*	IDH1|3417	0.196188	226.332	31.78732	**1.7E-05**
29	***RFC2|5982***	IDH1|3417	0.187794	1673.076	1.27817	**2.8E-09**
30	*FAM3C|10447*	IDH1|3417	0.108633	2101.338	0.45112	6.0E-01
31	*COMMD1|150684*	IDH1|3417	0.098073	-2338.74	938.3582	**1.2E-02**
32	*FAM32A|26017*	IDH1|3417	0.086003	467.2769	59.24544	**2.1E-03**
33	*FAM98C|147965*	IDH1|3417	0.081503	1432.472	102.0767	7.9E-01
34	*FAM131B|9715*	IDH1|3417	0.066649	2731.866	0.260898	**8.9E-03**

Table S7. KEGG pathways associated with the gene list from analysis 1 examining All gliomas IDH-wildtype.

No	Gene	Entrez identifier	KEGG_PATHWAY
1	DBF4 zinc finger(DBF4)	10926	Cell cycle
2	LLGL2, scribble cell polarity complex component(LLGL2)	3993	Hippo signaling pathway, Tight junction
3	TIMP metallopeptidase inhibitor 1(TIMP1)	7076	HIF-1 signaling pathway
4	cryptochrome circadian clock 2(CRY2)	1408	Circadian rhythm
5	eukaryotic translation initiation factor 4A3(EIF4A3)	9775	RNA transport, mRNA surveillance pathway, Spliceosome
6	glutaminyl-tRNA synthase (glutamine-hydrolyzing)-like 1(QRSL1)	55278	Aminoacyl-tRNA biosynthesis, Metabolic pathways
7	glyoxalase I(GLO1)	2739	Pyruvate metabolism
8	myosin heavy chain 7B(MYH7B)	57644	Tight junction
9	neuregulin 3(NRG3)	10718	ErbB signaling pathway
10	pyruvate carboxylase(PC)	5091	Citrate cycle (TCA cycle), Pyruvate metabolism, Metabolic pathways, Carbon metabolism, Biosynthesis of amino acids
11	replication factor C subunit 2(RFC2)	5982	DNA replication, Nucleotide excision repair, Mismatch repair

Table S8. KEGG pathways associated with the gene list from analysis 2 examining Primary GBM IDH-wildtype (GBM NR).

No	Gene	Entrez identifier	KEGG_PATHWAY
1	3-phosphoinositide dependent protein kinase 1(PDPK1)	5170	PPAR signaling pathway, FoxO signaling pathway, Sphingolipid signaling pathway, mTOR signaling pathway, PI3K-Akt signaling pathway, AMPK signaling pathway, Focal adhesion, T cell receptor signaling pathway, Fc epsilon RI signaling pathway, Neurotrophin signaling pathway, Insulin signaling pathway, Thyroid hormone signaling pathway, Insulin resistance, Aldosterone-regulated sodium reabsorption, Toxoplasmosis, Hepatitis C, Proteoglycans in cancer, Endometrial cancer, Prostate cancer, Non-small cell lung cancer, Choline metabolism in cancer
2	F-box protein 4(FBXO4)	26272	Ubiquitin mediated proteolysis
3	MPL proto-oncogene, thrombopoietin receptor(MPL)	4352	Cytokine-cytokine receptor interaction, Jak-STAT signaling pathway
4	collagen type IV alpha 6 chain(COL4A6)	1288	PI3K-Akt signaling pathway, Focal adhesion, ECM-receptor interaction, Protein digestion and absorption, Amoebiasis, Pathways in cancer, Small cell lung cancer
5	histone deacetylase 5(HDAC5)	10014	Alcoholism, Viral carcinogenesis
6	potassium voltage-gated channel subfamily Q member 5(KCNQ5)	56479	Cholinergic synapse
7	proteasome subunit alpha 3(PSMA3)	5684	Proteasome
8	protein phosphatase 1 catalytic subunit alpha(PPP1CA)	5499	mRNA surveillance pathway, cGMP-PKG signaling pathway, cAMP signaling pathway, Oocyte meiosis, Adrenergic signaling in cardiomyocytes, Vascular smooth muscle contraction, Hippo signaling pathway, Focal adhesion, Platelet activation, Long-term potentiation, Dopaminergic synapse, Inflammatory mediator regulation of TRP channels, Regulation of actin cytoskeleton, Insulin signaling pathway, Oxytocin signaling pathway, Insulin resistance, Amphetamine addiction, Alcoholism, Herpes simplex infection, Proteoglycans in cancer
9	sorting nexin 6(SNX6)	58533	Endocytosis
10	ubiquitin conjugating enzyme E2 F (putative)(UBE2F)	140739	Ubiquitin mediated proteolysis
11	ubiquitin protein ligase E3B(UBE3B)	89910	Ubiquitin mediated proteolysis
12	unc-51 like autophagy activating kinase 1(ULK1)	8408	Regulation of autophagy, mTOR signaling pathway
13	zinc finger E-box binding homeobox 1(ZEB1)	6935	MicroRNAs in cancer

Table S9. KEGG pathways associated with the gene list from analysis 3 examining Recurrent GBM IDH-wildtype (GBM R).

No	Gene	Entrez identifier	KEGG_PATHWAY
1	3-phosphoinositide dependent protein kinase 1(PDPK1)	5170	PPAR signaling pathway, FoxO signaling pathway, Sphingolipid signaling pathway, mTOR signaling pathway, PI3K-Akt signaling pathway, AMPK signaling pathway, Focal adhesion, T cell receptor signaling pathway, Fc epsilon RI signaling pathway, Neurotrophin signaling pathway, Insulin signaling pathway, Thyroid hormone signaling pathway, Insulin resistance, Aldosterone-regulated sodium reabsorption, Toxoplasmosis, Hepatitis C, Proteoglycans in cancer, Endometrial cancer, Prostate cancer, Non-small cell lung cancer, Choline metabolism in cancer
2	ATP binding cassette subfamily C member 5(ABCC5)	10057	ABC transporters
3	MFNG O-fucosylpeptide 3-beta-N-acetylglucosaminyltransferase(MFNG)	4242	Other types of O-glycan biosynthesis, Notch signaling pathway
4	ectodysplasin A(EDA)	1896	Cytokine-cytokine receptor interaction
5	histone deacetylase 5(HDAC5)	10014	Alcoholism, Viral carcinogenesis
6	myosin heavy chain 15(MYH15)	22989	Tight junction
7	period circadian clock 3(PER3)	8863	Circadian rhythm, Circadian entrainment, Herpes simplex infection
8	platelet derived growth factor subunit A(PDGFA)	5154	MAPK signaling pathway, Ras signaling pathway, Rap1 signaling pathway, PI3K-Akt signaling pathway, Focal adhesion, Gap junction, Regulation of actin cytoskeleton, HTLV-I infection, Pathways in cancer, Transcriptional misregulation in cancer, MicroRNAs in cancer, Glioma, Prostate cancer, Melanoma, Choline metabolism in cancer
9	polycomb group ring finger 3(PCGF3)	10336	Signaling pathways regulating pluripotency of stem cells
10	proteasome subunit alpha 3(PSMA3)	5684	Proteasome
11	replication factor C subunit 2(RFC2)	5982	DNA replication, Nucleotide excision repair, Mismatch repair
12	unc-51 like autophagy activating kinase 1(ULK1)	8408	Regulation of autophagy, mTOR signaling pathway

Table S10. Results for the survival analyses for validation of the prognostic biomarkers in five independent datasets. Results list the *P*-values from either the Log-rank or the Wilcoxon tests.

Dataset/Reference	GBM / GBM IDH-wildtype	Data platform	TSPYL2	JAKMIP1	CIT	TMTC1
TCGA-GBM[8]	GBM IDH-wildtype	RNA-seq	*P*=0.0359	*P*=0.0376	*P*=0.0441	*P*=0.0281
CGGA; Zhao[37]	GBM IDH-wildtype	RNA-seq	NS	NS	NS	NS
Gravendeel[30]	GBM IDH-wildtype	Microarray	NS	NS	-	NS
Rembrandt; Madhavan[31]	GBM	Microarray	NS	NS	NS	*P*=0.0069
Lee[38]	GBM	Microarray	NS	-	-	-
Murat[34]	GBM	Microarray	NS	NS	-	NS

NS – Non-significant; "-" – Not applicable because the gene was not represented on the microarray

Table S11. List of the proteins (n=202) correlated with IDH1 mRNA expression using a 50% quartile cut-off.

**Protein**	**Average Low**	**Average High**	***P*-value**	**Adjusted *P*-value**
EGFR	0.0793	1.26	0.00142	0.288
Bax	0.021	0.362	0.00228	0.463
S6	0.0302	0.346	0.00884	1
Chk2_pT68	0.00191	-0.156	0.012	1
eEF2K	-0.031	0.257	0.0137	1
GATA6	0.0566	-0.112	0.0155	1
N.Cadherin	0.0224	0.202	0.016	1
Stathmin	0.113	-0.0513	0.0201	1
Rictor_pT1135	0.0111	-0.0993	0.0204	1
Cyclin_D1	0.0809	-0.104	0.0207	1
EGFR_pY1068	0.468	1.59	0.0259	1
IRS1	0.0406	-0.104	0.0273	1
beta.Catenin	-0.185	0.177	0.0284	1
p53	0.145	-0.0475	0.0311	1
VEGFR2	0.0843	-0.183	0.0343	1
HER3_pY1289	0.0457	-0.117	0.0363	1
YAP_pS127	-0.00597	0.252	0.0363	1
Cyclin_E2	0.0178	-0.0702	0.0375	1
Caveolin.1	0.281	-0.281	0.0399	1
p38_pT180_Y182	-0.108	0.205	0.0403	1
P.Cadherin	0.0309	0.227	0.0425	1
p70S6K_pT389	0.315	-0.128	0.0442	1
NDRG1_pT346	0.0563	-0.324	0.0487	1
Smad1	-0.031	0.182	0.0574	1
MEK1	0.0947	-0.126	0.0616	1
Bcl.2	-0.0317	0.188	0.065	1
EGFR_pY1173	0.428	1.13	0.066	1
Chk1	0.0137	-0.0953	0.0724	1
eIF4E	-0.00367	0.0926	0.073	1
ASNS	0.121	-0.0965	0.0775	1
Lck	-0.0964	0.0688	0.0817	1
E.Cadherin	0.128	0.332	0.0827	1
AMPK_alpha	-0.0657	0.0667	0.0853	1
Mre11	0.0125	-0.0677	0.0887	1
PRDX1	-0.00623	0.126	0.0899	1
14.3.3_zeta	-0.0201	-0.133	0.102	1
SLC1A5	0.177	-0.00898	0.102	1
Src	-0.03	-0.151	0.103	1
Smac	0.159	-0.034	0.106	1
p38_MAPK	-0.0548	0.114	0.106	1
C.Raf_pS338	0.064	-0.0667	0.107	1
Rictor	0.00474	-0.145	0.108	1
TAZ	0.126	-0.0444	0.114	1

AMPK_pT172	-0.197	0.0662	0.126	1
c.Kit	0.37	-0.0936	0.128	1
C.Raf	0.00823	-0.0953	0.132	1
S6_pS240_S244	0.0681	0.35	0.132	1
Notch1	-0.0599	0.13	0.142	1
Beclin	0.0597	-0.0747	0.146	1
Annexin.1	-0.0103	0.28	0.151	1
4E.BP1_pT70	0.0168	-0.0514	0.152	1
HER2_pY1248	0.155	0.599	0.156	1
Rad50	0.0121	0.119	0.156	1
Rb	0.0428	0.171	0.164	1
Bcl.xL	-0.032	0.0636	0.166	1
eEF2	-0.0229	0.0813	0.18	1
JNK_pT183_pY185	0.0528	-0.163	0.185	1
HER2	-0.0546	0.116	0.192	1
ER.alpha_pS118	0.132	0.0612	0.194	1
HSP70	0.0836	-0.245	0.206	1
Akt_pS473	-0.018	-0.29	0.207	1
TIGAR	-0.0405	-0.0998	0.218	1
MEK1_pS217_S221	0.0534	-0.0915	0.227	1
PDCD4	-0.0533	0.0521	0.227	1
GATA3	0.0976	-0.00999	0.236	1
MSH2	0.0771	0.216	0.239	1
PCNA	0.0235	0.125	0.24	1
S6_pS235_S236	0.0813	0.277	0.243	1
c.Met_pY1235	-0.00146	-0.0529	0.249	1
INPP4B	0.221	0.0798	0.264	1
STAT5.alpha	-0.138	0.014	0.274	1
53BP1	-0.14	0.109	0.275	1
BRCA2	0.0814	-0.0286	0.275	1
LKB1	-0.0113	-0.0711	0.283	1
Bad_pS112	-0.0772	-0.143	0.285	1
XRCC1	0.0694	0.12	0.285	1
Cyclin_E1	0.082	0.000234	0.287	1
PDK1_pS241	-0.0148	-0.146	0.288	1
Bim	0.148	0.312	0.291	1
4E.BP1_pS65	0.00495	-0.0661	0.319	1
G6PD	-0.0162	0.0821	0.352	1
ER.alpha	0.105	0.269	0.356	1
PREX1	-0.0525	0.139	0.356	1
ERK2	-0.0521	-0.229	0.368	1
Collagen_VI	0.0553	-0.0587	0.383	1
NF.kB.p65_pS536	-0.044	0.11	0.383	1
p90RSK	-0.126	-0.201	0.39	1
Heregulin	0.0322	-0.0468	0.394	1
MAPK_pT202_Y204	0.0514	-0.166	0.404	1
NF2	-0.0942	0.0133	0.414	1
PKC.alpha	-0.0688	0.0932	0.417	1

PEA15	0.166	0.0174	0.419	1
GAPDH	0.103	-0.146	0.421	1
4E.BP1_pT37_T46	0.0739	0.23	0.439	1
GAB2	0.0197	0.144	0.445	1
p27_pT157	-0.00042	-0.0381	0.452	1
Claudin.7	0.035	0.117	0.456	1
CDK1_pY15	0.155	0.0881	0.457	1
STAT3_pY705	0.0198	0.135	0.459	1
Dvl3	-0.0213	0.0329	0.47	1
PDK1	0.000426	-0.0408	0.47	1
JNK2	-0.0459	-0.101	0.473	1
BRD4	0.000225	0.101	0.479	1
ARID1A	0.0227	-0.0235	0.486	1
PKC.delta_pS664	0.0208	0.121	0.491	1
TFRC	-0.019	0.121	0.506	1
SETD2	0.0313	-0.0202	0.507	1
CD20	0.0623	0.0209	0.513	1
YB.1_pS102	-0.0923	-0.0363	0.523	1
TSC1	-0.0595	0.0165	0.526	1
Smad4	0.0345	0.0844	0.532	1
ACC_pS79	-0.0715	0.0349	0.535	1
Axl	-0.0336	0.035	0.535	1
Chk2	0.0516	0.116	0.537	1
GSK3_pS9	-0.0682	-0.162	0.538	1
Paxillin	0.00207	0.0545	0.538	1
MYH11	0.517	0.326	0.54	1
Ku80	0.000871	-0.0755	0.541	1
p90RSK_pT359_S363	0.0281	0.0801	0.541	1
IGFBP2	0.0842	0.217	0.545	1
4E.BP1	0.096	0.149	0.548	1
Rb_pS807_S811	0.203	0.0786	0.551	1
eIF4G	-0.0251	0.0412	0.559	1
XBP1	0.202	0.101	0.56	1
Src_pY416	0.0341	0.15	0.563	1
PAI.1	0.332	0.171	0.58	1
Chk1_pS345	0.0253	0.0563	0.583	1
Caspase.8	-0.00181	0.0435	0.584	1
c.Jun_pS73	0.0876	0.139	0.584	1
cIAP	0.0208	-0.0262	0.589	1
mTOR	-0.0389	-0.134	0.604	1
Rab11	-0.00259	-0.0514	0.608	1
Myosin.IIa_pS1943	-0.0262	0.0466	0.619	1
ACVRL1	0.146	0.213	0.623	1
PEA15_pS116	0.0044	0.0659	0.624	1
DJ.1	-0.0343	0.0232	0.627	1
HER3	0.0996	0.00876	0.627	1
Tuberin	-0.136	-0.24	0.627	1
SF2	0.0045	-0.0322	0.639	1

CDK1	0.04	0.087	0.645	1
Akt_pT308	-0.0669	0.0222	0.649	1
ETS.1	-0.00085	0.047	0.65	1
Akt	-0.0745	0.017	0.659	1
PARP_cleaved	0.0983	0.168	0.667	1
Rad51	-0.0395	-0.0646	0.671	1
N.Ras	0.0305	-0.0111	0.677	1
FASN	0.0681	0.13	0.68	1
A.Raf_pS299	0.0784	0.113	0.684	1
ARHI	0.0721	0.145	0.684	1
Syk	-0.00868	-0.0836	0.684	1
Rab25	0.0564	0.0968	0.687	1
Src_pY527	-0.0431	-0.105	0.692	1
CD31	0.0518	-7.7E-05	0.693	1
B.Raf	-0.0667	-0.126	0.696	1
MSH6	0.0653	-0.00164	0.697	1
p21	-0.0164	0.00598	0.698	1
RBM15	-0.0806	-0.142	0.723	1
Cyclin_B1	0.402	0.344	0.73	1
Bid	0.0601	0.0853	0.735	1
c.Myc	0.00429	-0.0204	0.741	1
Smad3	0.00768	0.028	0.742	1
Bak	0.0329	0.0614	0.747	1
p27_pT198	0.0301	0.0124	0.748	1
AR	0.0906	0.116	0.765	1
PR	0.142	0.189	0.77	1
PI3K.p110.alpha	-0.033	-0.0597	0.772	1
FoxM1	0.136	0.162	0.784	1
Acetyl.a.Tubulin.Lys40	-0.0651	-0.152	0.794	1
ERCC1	-0.00769	-0.0193	0.804	1
VHL	-0.0102	0.0287	0.808	1
Myosin.IIa	-0.126	-0.147	0.812	1
p62.LCK.ligand	0.123	0.0886	0.814	1
Annexin_VII	0.0596	0.0384	0.831	1
Snail	0.0654	0.0876	0.831	1
14.3.3_epsilon	-0.0288	-0.0387	0.839	1
YB.1	-0.00561	-0.0248	0.84	1
PTEN	-0.0596	-0.0392	0.841	1
SCD	0.0354	0.0515	0.848	1
GSK3.alpha.beta	-0.0183	-0.0412	0.866	1
MIG.6	0.0206	0.0114	0.877	1
GSK3.alpha.beta_pS21_S9	-0.074	-0.0977	0.878	1
Tuberin_pT1462	-0.0273	-0.0132	0.888	1
Transglutaminase	0.044	0.0521	0.905	1
CD49b	0.102	0.114	0.911	1
Raptor	-0.0398	-0.0519	0.912	1
14.3.3_beta	0.0677	0.0826	0.913	1
YAP	0.102	0.116	0.919	1

PKC.alpha_pS657	-0.0379	-0.0146	0.92	1
Fibronectin	0.116	0.0965	0.922	1
Bap1.c.4	0.0166	0.0109	0.933	1
Caspase.7_cleavedD198	0.0984	0.109	0.938	1
p70S6K	-0.0353	-0.0434	0.939	1
Shc_pY317	0.0488	0.0562	0.94	1
PRAS40_pT246	-0.0797	-0.075	0.95	1
p27	0.101	0.0925	0.95	1
ATM	-0.00395	-0.0201	0.951	1
mTOR_pS2448	-0.0362	-0.0316	0.953	1
ACC1	-0.0644	-0.0733	0.968	1
PI3K.p85	-0.0373	-0.041	0.968	1
FOXO3a_pS318_S321	-0.0293	-0.0303	0.979	1
c.Met	-0.0173	-0.016	0.988	1
FOXO3a	0.0842	0.0835	0.996	1
PKC.pan_BetaII_pS660	-0.155	-0.154	0.996	1

References

8. Cancer Genome Atlas (TCGA) Research Network. Comprehensive genomic characterization defines human glioblastoma genes and core pathways. *Nature* **2008**, 455(7216), 1061-1068.

30. Gravendeel, L. A., Kouwenhoven, M. C., Gevaert, O., de Rooi, J. J., Stubbs, A. P., Duijm, J. E., ... & French, P. J. Intrinsic gene expression profiles of gliomas are a better predictor of survival than histology. *Cancer research* **2009**, 69(23), 9065-9072.

31. Madhavan, S., Zenklusen, J. C., Kotliarov, Y., Sahni, H., Fine, H. A., & Buetow, K. Rembrandt: helping personalized medicine become a reality through integrative translational research. *Molecular cancer research* **2009**, 7(2), 157-167.

32. Kamoun, A., Idbaih, A., Dehais, C., Elarouci, N., Carpentier, C., Letouzé, E., Colin, C., Mokhtari, K., Jouvet, A., Uro-Coste, E., Martin-Duverneuil, N., Sanson, M., Delattre, J. Y., Figarella-Branger, D., de Reyniès, A., Ducray, F., & POLA network. Integrated multi-omics analysis of oligodendroglial tumours identifies three subgroups of 1p/19q co-deleted gliomas. Nature communications **2016**, 7, 11263.

33. Grzmil M, Morin P Jr, Lino MM, Merlo A, Frank S, Wang Y, Moncayo G, Hemmings BA. MAP kinase-interacting kinase 1 regulates SMAD2-dependent TGF-β signaling pathway in human glioblastoma. Cancer Res. **2011** 71(6):2392-402.

34. Murat, A., Migliavacca, E., Gorlia, T., Lambiv, W. L., Shay, T., Hamou, M. F., ... & Hegi, M. E. Stem cell-related" self-renewal" signature and high epidermal growth factor receptor expression associated with resistance to concomitant chemoradiotherapy in glioblastoma. *Journal of clinical oncology* **2008**, 26(18), 3015-3024.

35. Puchalski, R.B.; Shah, N.; Miller, J.; Dalley, R.; Nomura, S.R.; Yoon, J.G.; Foltz, G.D. An anatomic transcriptional atlas of human glioblastoma. *Science* **2018**, 360, 660–663.

37. Zhao, Z.; Meng, F.; Wang, W.; Wang, Z.; Zhang, C.; Jiang, T. Comprehensive RNA-seq transcriptomic profiling in the ma-lignant progression of gliomas. Sci. Data 2017, 4, 1–7.

38. Lee, Y., Scheck, A. C., Cloughesy, T. F., Lai, A., Dong, J., Farooqi, H. K., ... & Nelson, S. F. Gene expression analysis of glioblastomas identifies the major molecular basis for the prognostic benefit of younger age. *BMC medical genomics* **2008**, 1(1), 1-12.
